# An Oligomer Approach for Blue Thermally Activated Delayed Fluorescent Emitters Based on Twisted Donor–Acceptor Units

**DOI:** 10.1021/acs.chemmater.2c03438

**Published:** 2023-02-28

**Authors:** Eimantas Duda, Subeesh Madayanad Suresh, David Hall, Sergey Bagnich, Rishabh Saxena, David B. Cordes, Alexandra M. Z. Slawin, David Beljonne, Yoann Olivier, Anna Köhler, Eli Zysman-Colman

**Affiliations:** † Soft Matter Optoelectronics, BIMF & BPI, University of Bayreuth, Universitätsstraße 30, 95447 Bayreuth, Germany; ‡ Organic Semiconductor Centre, EaStCHEM School of Chemistry, University of St Andrews, St Andrews KY16 9ST, UK; § Laboratory for Chemistry of Novel Materials, Materials Research Institute, University of Mons, Place du Parc 20, 7000 Mons, Belgium; ∥ Unité de Chimie Physique Théorique et Structurale & Laboratoire de Physique du Solide, Namur Institute of Structured Matter, Université de Namur, Rue de Bruxelles, 61, 5000 Namur, Belgium

## Abstract

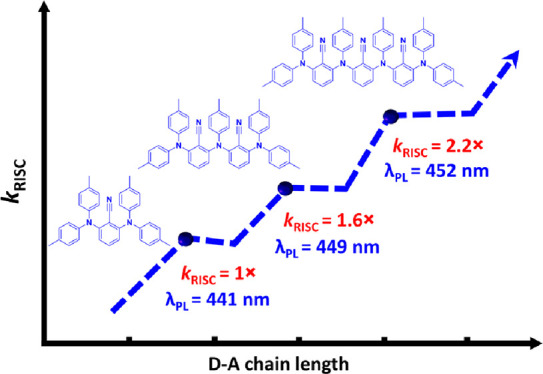

The development of efficient blue donor–acceptor thermally activated delayed fluorescence (TADF) emitters remains a challenge. To enhance the efficiency of TADF-related processes of the emitter, we targeted a molecular design that would introduce a large number of intermediate triplet states between the lowest energy excited triplet (T_1_) and singlet (S_1_) excited states. Here, we introduce an oligomer approach using repetitive donor–acceptor units to gradually increase the number of quasi-degenerate states. In our design, benzonitrile (BN) moieties were selected as acceptors that are connected together via the amine donors, acting as bridges to adjacent BN acceptors. To preserve the photoluminescence emission wavelength across the series, we employed a design based on an *ortho* substitution pattern of the donors about the BN acceptor that induces a highly twisted conformation of the emitters, limiting the conjugation. Via a systematic photophysical study, we show that increasing the oligomer size allows for enhancement of the intersystem crossing and reverse intersystem crossing rates. We attribute the increasing intersystem crossing rate to the increasing number of intermediate triplet states along the series, confirmed by the time-dependent density functional theory. Overall, we report an approach to enhance the efficiency of TADF-related processes without changing the blue photoluminescence color.

## Introduction

Organic light-emitting diode (OLED) technology enables thin, lightweight, flexible, and highly efficient displays and lighting products that are not readily achievable with other technologies.^1−3^ Current research in OLED materials has mainly focused on the development of efficient purely organic thermally activated delayed fluorescence (TADF) emitters that show comparable exciton utilization efficiencies to commercially used yet unsustainable red- and green-emitting iridium(III) complexes and surpass those of blue, fluorescent compounds.^3,4^ TADF emitters can thermally up-convert non-emissive triplet excitons (T_1_) to emissive singlet excitons (S_1_) through reverse intersystem crossing (RISC), and the efficiency of this process is governed in part by the singlet-triplet energy gap, Δ*E*_ST_, and the magnitude of the spin orbit coupling (SOC) between singlet and triplet states.^5,6^ According to El-Sayed’s rule, the transition between singlet and triplet states possessing the same nature is not efficient.^7,8^ A possible way to increase the SOC between the singlet and triplet manifolds in the compound is for RISC to proceed via intermediate triplet states of different character to S_1_, frequently of a stronger locally excited (LE) character, that can vibronically couple to T_1_.^9−11^ There are a small number of strategies documented that achieve a higher number of intermediate triplet states in donor–acceptor (D-A) TADF emitters, associated with a larger number of either donor or acceptor units.^12−16^ In principle, a higher number of repetitive units should result in a larger number of quasi-degenerate states that also produces a higher number of intermediate triplet states.^12^

**Figure 1:**
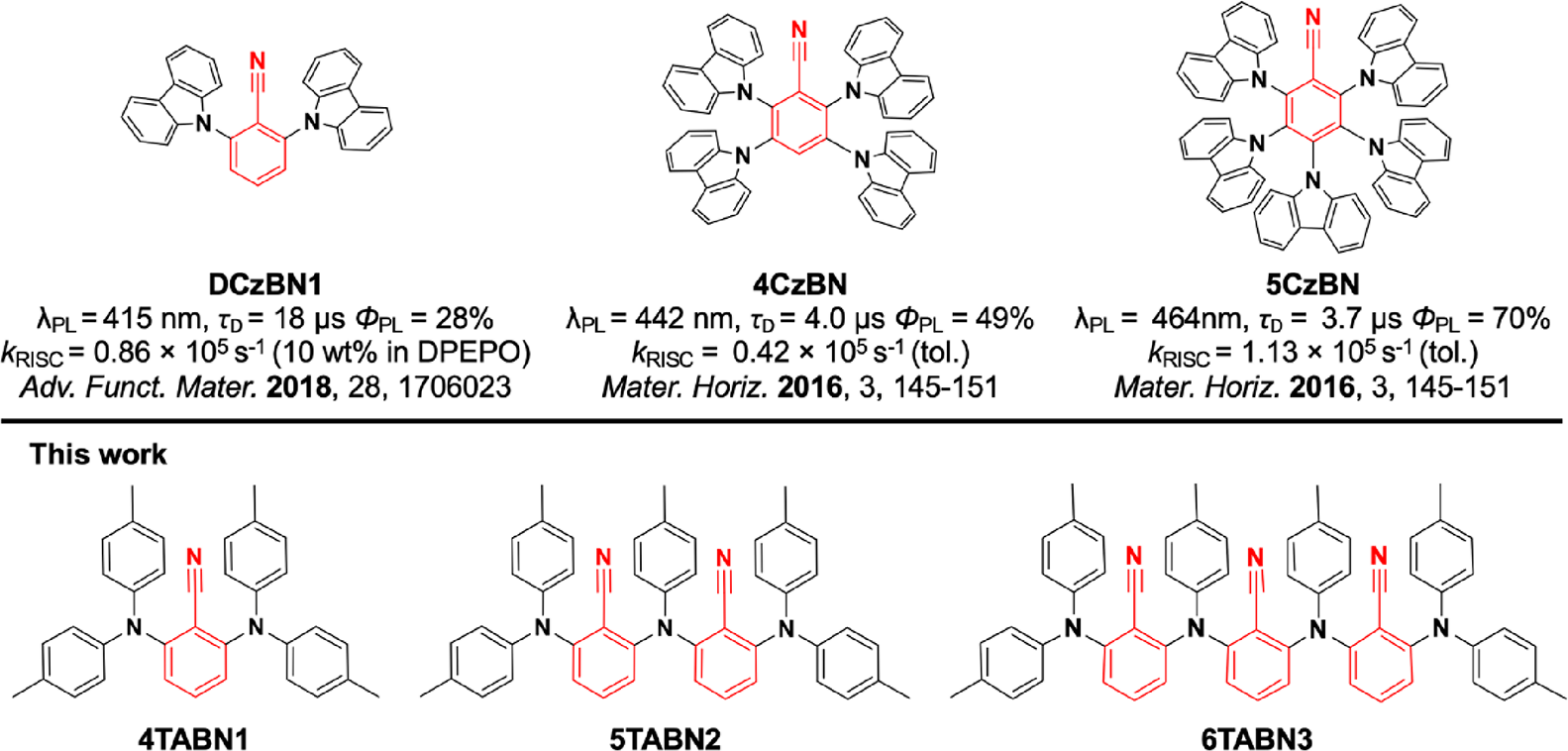
Chemical structures of the literature compounds and the compounds investigated in this work.

One known design strategy to produce a system that possesses intermediate triplet states is based on a multidonor to a single acceptor system like poly(carbazole)-substituted benzonitrile (BN) derivatives.^12,13^ For example, time-dependent density functional theory (TD-DFT) calculations predict that the number of intermediate triplet states increases from two to three when the number of donor carbazoles is increased from four in 2,3,5,6-tetra(9*H*-carbazol-9-yl)benzonitrile (**4CzBN**) to five in 2,3,4,5,6-penta-(9*H*-carbazol-9-yl)benzonitrile (**5CzBN**) (Figure 1).^17^ This is reflected in experimentally faster RISC, *k*_RISC_, and smaller singlet triplet gaps, Δ*E*_ST_, with increasing number of carbazole donors from **DCzBN1** to **4CzBN** and **5CzBN**, while also attractively showing enhanced photoluminescence quantum yields, Φ_PL_.

Another strategy to ensure a larger number of intermediate triplet states is to increase the number of donors and acceptor repeat units within a D-A main chain polymer.^18^ A rare example of such kind of a polymer was reported by Adachi and co-workers.^14^ While the group demonstrated solution-processed OLEDs that perform well using TADF-active polymers as emitters based on this design concept, the number of intermediate triplet states in their polymers was actually not investigated, either theoretically or experimentally through a photophysical study. Furthermore, this fine-tuning of the TADF properties resulted in a strong red shift of photoluminescence.^14^

To fine-tune the TADF-related parameters without invoking an undesired red shift of the photoluminescence, here we present a new strategy to ensure that a large number of intermediate triplet states is present in the emitter, resulting in fast *k*_RISC_. We report a family of D-A TADF emitters that have BN as the acceptor unit that are connected via the amine donors *ortho* to the nitrile acceptor, acting as bridges to adjacent BN acceptors (Figure 1). Our reference emitter **4TABN1** is a ditolyl amine version of the previously reported carbazole-benzonitrile based **DCzBN1** (a blue TADF emitter). The structural design of our longer emitters is reminiscent of an alternating D-A main chain polymer. In this report, we targeted blue emission in short D-A oligomers through the selection of tolylamine and BN moieties as weak D and A units, respectively. The number of donor units attached to a single BN motif is limited to two. The *ortho* substitution pattern of the donors translates to highly twisted conformations of the emitters, which should limit the interaction between hole and electron densities to ensure a small Δ*E*_ST_. Furthermore, a highly twisted conformation should prevent charge delocalization across the molecular structure and thus the undesired red shift of the emission.

Here, we find that increasing the molecular chain length with repetitive D-A units leads to a gradual increase in the number of intermediate triplet states. As a consequence of the greater number of intermediate triplet states, the RISC-related parameters such as the intersystem crossing rate constant, *k*_ISC_, as well as *k*_RISC_ increase with increasing chain length. Most importantly, our approach provides a way to increase the RISC-related parameters while simultaneously preserving the emission wavelength.

## Experimental Methods

### General Synthetic Procedures

All commercially available chemicals and reagent-grade solvents were used as received. Solvents used in the reactions were dry and deaerated using a solvent purification system. Air-sensitive reactions are done under a nitrogen atmosphere using Schlenk techniques. Flash column chromatography was carried out using silica gel (Silia-P from Silicycle, 60 Å, 40–63 μm). Analytical thin-layer chromatography (TLC) was performed with silica plates with aluminum backings (250 μm with an F-254 indicator). TLC visualization was accomplished by use of a 254/365 nm UV lamp. ^1^H and ^13^C and NMR spectra were recorded on a Bruker Advance spectrometer (400 or 500 MHz for ^1^H and 100 or 125 MHz for ^13^C). The following abbreviations have been used for multiplicity assignments: “s” for singlet, “d” for doublet, “t” for triplet, “dd” for doublet of doublets, “td” for triplet of doublets, and “m” for multiplets. Deuterated acetone (acetone-d6), chloroform (CDCl_3_), and DMSO (DMSO-d6) were used as the solvents of recording NMR. ^1^H NMR spectra were referenced to the corresponding solvent peak. HPLC analysis was conducted on a Shimadzu Nexera series HPLC system. HPLC traces were performed using an ACE Excel 2 C18 (3 μm C18, 3.0 × 150 mm) analytical reverse phase column using a methanol–water mobile phase. GCMS analyses were carried out in a QP2010 SE system. Melting points were measured using open-ended capillaries on a melting point apparatus IA9200 and are uncorrected. High-resolution mass spectrometry (HRMS) was performed by the Mass Spectrometry Facility, University of St Andrews. Elemental analysis were performed using the facility of London Metropolitan University.

### Thermogravimetric Analysis

Analysis was carried out by heating the sample from 25 to 700 °C. Measurements were done under a nitrogen atmosphere at a heating rate of 10 °C/min on a Netzsch 409 C instrument. Decomposition temperature (*T*_d_) is defined as the temperature at which 5% of the material is lost.

### Electrochemistry Measurements

Cyclic voltammetry (CV) and differential pulse voltammetry (DPV) analyses were performed on an Electrochemical Analyzer potentiostat model 620E from CH Instruments at a sweep rate of 100 mV/s. DPV was conducted with an increment potential of 0.004 V and a pulse amplitude, width, and period of 50 mV, 0.05, and 0.5 s, respectively. Samples were prepared in HPLC-grade acetonitrile (MeCN) solutions, which were degassed by sparging with MeCN-saturated nitrogen gas for 5 min prior to all measurements. All measurements were performed using 0.1 M tetra-*n*-butylammonium hexafluorophosphate, [_*n*_Bu_4_N]PF_6_, in MeCN. An Ag/Ag^+^ electrode was used as the reference electrode, a glassy carbon electrode was used as the working electrode, and a platinum wire was used as the counter electrode. The redox potentials are reported relative to a saturated calomel electrode (SCE) with a ferrocene/ferrocenium (Fc/Fc^+^) redox couple as the internal standard (0.38 V vs SCE).^19^ The HOMO and LUMO energy levels were determined using the relation *E*_HOMO/LUMO_ = −(*E*^ox/red^ + 4.8) eV,^20^ where *E*^ox^ and *E*^red^ are the anodic and cathodic DPV peak potentials of the first oxidation and reduction peaks, respectively.

### Photophysical Measurements

Solutions were prepared in HPLC-grade oxygen-free toluene at a concentration of 0.05 mg/mL, equal to 1.0 × 10^–4^, 7.15 × 10^–5^, and 5.52 × 10^–5^ M for **4TABN1**, **5TABN2**, and **6TABN3**, respectively. Neat and doped films were prepared from a 2.5 mg/mL oxygen-free chloroform solution by spin-coating at 2000 RPM. For the doped films, 1, 3, 10, or 30 wt % of guest had been dispersed into DPEPO. For photoluminescence quantum yield (PLQY) measurements, drop-cast films were made at 60 °C from a solution with the oxygen-free chloroform. Absorption measurements were carried out at room temperature using a Cary 5000 UV–vis–NIR absorption spectrophotometer. Steady-state photoluminescence spectra at RT and 77 K, PL emission in the ms range at 77 K, and PLQY measurements at RT were obtained using the JASCO FP-8600 spectrofluorometer for different excitations (as specified in figure captions). The thin-film PLQY was measured in a N_2_-filled integrating sphere. For measurements at 77 K, the film or cuvette was immersed in liquid nitrogen. The time-resolved photoluminescence measurements were obtained using an iCCD camera and exponentially increasing delay and gating times, where the gating time is kept lower by a factor of 10 compared to the delay time. Samples were excited at 355 nm by a q-switched laser from QS Lasers (MPL15100-DP). Emission from the samples was focused onto a spectrograph (Oriel MS257) and detected on a gated iCCD camera (iStar A-DH334T-18F-03). The measurements were recorded under vacuum or in a He-filled atmosphere.

For the rate constant and quantum yield calculations, the approach is based on refs^21,22^ and is summarized below.
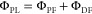
1where Φ_PF_ and Φ_DF_ are the prompt and delayed photoluminescence quantum yields, respectively.
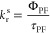
2where *k*_r_^s^ is the radiative decay rate constant of the singlet state, Φ_PF_ is the prompt emission quantum yield, and τ_PF_ is the prompt emission lifetime.

In our case*.*, Φ_DF_/Φ_PF_ < 4 and Φ_RISC_ cannot be approximated to unity, so Φ_ISC_ must be evaluated through a linear fit by plotting τ_DF_ versus Φ_DF_/Φ_PF_ at different temperatures.
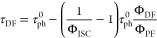
3where τ^0^_ph_ is the phosphorescence lifetime.
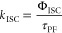
4where *k*_ISC_ is the intersystem crossing rate constant.
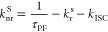
5where *k*^S^_nr_ is the non-radiative decay rate constant from the singlet state.
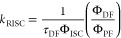
6where *k*_RISC_ is the reverse intersystem rate constant.

### Quantum Chemical Calculations

The density functional theory calculations were performed with the Gaussian 09 revision D.018 suite.^23^ Ground-state optimized structures were calculated starting from GaussView drawn structures using the PBE0 functional^24^ and the 6-31G(d,p) basis set^25^ or the M062X functional^26^ and the 6-31G(d,p) basis set. Vertical excited-state calculations were performed within the Tamm–Dancoff approximation (TDA)^27^ at the same level of theory as the ground-state optimization. Optimized singlet excited states were obtained using TDA-PBE0/6-31G(d,p) in the gas phase. The attachment/detachment formalism was employed to calculate Φ_s_ values for each of the excited states using the NANCY package,^28^ with a value of 0.00–0.32 representing a pure charge transfer (CT) state, 0.33–0.66 representing a mixed state, coined Mix, and 0.67–1.00 representing an LE state. Spin-orbit coupling matrix elements between the S_1_ state and each of the T_1_–T_4_ states were calculated using PySOC at the TDA-PBE0/6-31G(d,p) level in the gas phase^29^ using the ground-state optimized geometry. Molecular orbitals were visualized using GaussView 6.0.^30^

### X-ray Crystallography

Crystals for **4TABN1** and **5TABN2** were obtained by slow diffusion of pentane vapor into a saturated toluene (**4TABN1**) or acetone (**5TABN2**) solution of the compounds at room temperature over several days. X-ray diffraction data for compound **6** were collected using a Rigaku FR-X Ultrahigh Brilliance Microfocus RA generator/confocal optics with an XtaLAB P200 diffractometer [Mo Kα radiation (λ = 0.71073 Å)]. Diffraction data for compound **6** were collected using a Rigaku MM-007HF High Brilliance RA generator/confocal optics with an XtaLAB P100 diffractometer [Cu Kα radiation (λ = 1.54187 Å)]. Intensity data were collected at 173 K, using either just ω steps or both ω and φ steps, accumulating area detector images spanning at least a hemisphere of reciprocal space. Data for both compounds were collected using CrystalClear^31^ and processed (including correction for Lorentz, polarization, and absorption) using CrysAlisPro.^32^ Structures were solved by direct methods (SIR2011)^33^ and refined by full-matrix least squares against F^2^ (SHELXL-2018/3).^34^ Non-hydrogen atoms were refined anisotropically, and hydrogen atoms were refined using a riding model. Both structures showed a void space containing poorly ordered solvent molecules (**4TABN1**: 727 Å^3^, toluene; **5TABN2**: 1068 Å^3^, acetone), and the SQUEEZE^35^ routine implemented in PLATON^36^ was used to remove the contribution to the diffraction pattern of the electron density in the void spaces. All calculations except SQUEEZE were performed using the CrystalStructure^37^ interface. Selected crystallographic data are presented in Table S8. CCDC 2218300–2218301 contains the supplementary crystallographic data for this paper. These data can be obtained free of charge from The Cambridge Crystallographic Data Centre via www.ccdc.cam.ac.uk/structures.

## Results and Discussion

**Figure 2:**
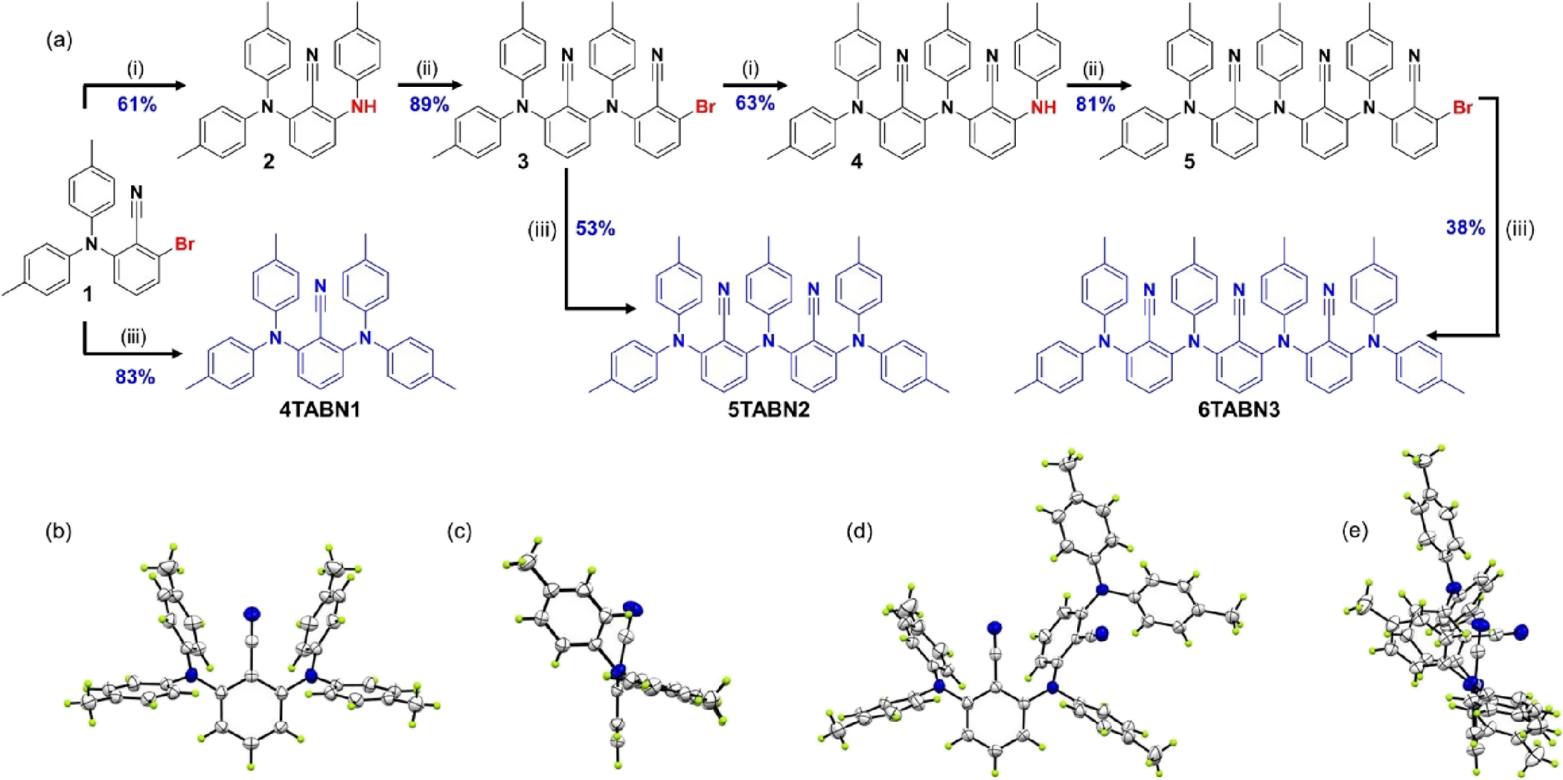
(a) Synthetic route for the target emitters and thermal ellipsoid diagrams of emitters obtained from single-crystal X-ray analysis; (b) and (c) are the plane and side views of **4TABN1**; (d) and (e) are the plane and side views of **5TABN2**. Thermal ellipsoids are displayed at the 50% probability level, and one orientation of disordered methyl hydrogens in **4TABN1** is omitted for clarity. (i) *p*-toluidine, Pd_2_(dba)_3_, SPhos, NaO^*t*^Bu, toluene, 110 °C, 12 h; (ii) 2-bromo-6-fluorobenzonitrile, Cs_2_CO_3_, DMF, 140 °C, 12 h; (iii) di-*p*-tolylamine, Pd_2_(dba)_3,_ SPhos, NaO^*t*^Bu, toluene, 110 °C, 12 h.

The emitter, **4TABN1**, was obtained from a two-step sequential set of SNAr and Buchwald–Hartwig cross-coupling reactions in good yields (Figure 2). By following this synthetic protocol iteratively, **5TABN2** and **6TABN3** were obtained from intermediate **1** in three and five steps, respectively. Due to the lower reactivity of CN-substituted amine fragments such as **2** and **4**, coupling reactions between **1** and **2** or **3** and **4** were not observed to proceed efficiently using the conditions given in Figure 2. The structural identity and purity of the three emitters were established from a combination of ^1^H and ^13^C NMR spectroscopy, HRMS, HPLC and elemental analyses, and single crystal X-ray analysis. All three emitters possess excellent solubility in common organic solvents such as acetone, acetonitrile, DCM, chloroform, THF, and DMF. Single-crystal XRD analysis was carried out to provide insight into the conformation of the compounds and any potential intermolecular interactions in the solid state. Single crystals were grown by slow diffusion of hexane vapors into an acetone solution of the emitters. **4TABN1** and **5TABN2** both possess highly twisted conformations in the solid state (Figure 2). There are no intermolecular π-stacking interactions in the unit cell.

To determine the energies of the HOMO and LUMO levels, we performed CV and DPV measurements (Figure S28). The corresponding HOMO energy levels extracted from the peak value of the first oxidation wave (*E*^ox^ = 0.95, 0.99, and 1.0 V vs SCE) of the DPVs were found to stabilize only slightly along the series, being −5.75, −5.79, and −5.80 eV for **4TABN1**, **5TABN2**, and **6TABN3**, respectively. The LUMO levels were determined from the peak value of the first reduction wave of the DPVs (*E*^red^ = −2.25, −2.17, and – 2.14 V vs SCE) and correspond to −2.55, −2.63, and – 2.66 eV, respectively, and are also stabilized slightly along the series. The corresponding energy gaps were likewise found to decrease slightly at 3.20, 3.16, and 3.14 eV, respectively, for **4TABN1**, **5TABN2**, and **6TABN3** as a function of an increasing number of repeat units. The trends in both progressively stabilizing HOMO and LUMO align well with the DFT calculated trends (vide infra).

### Photophysical Properties

**Figure 3:**
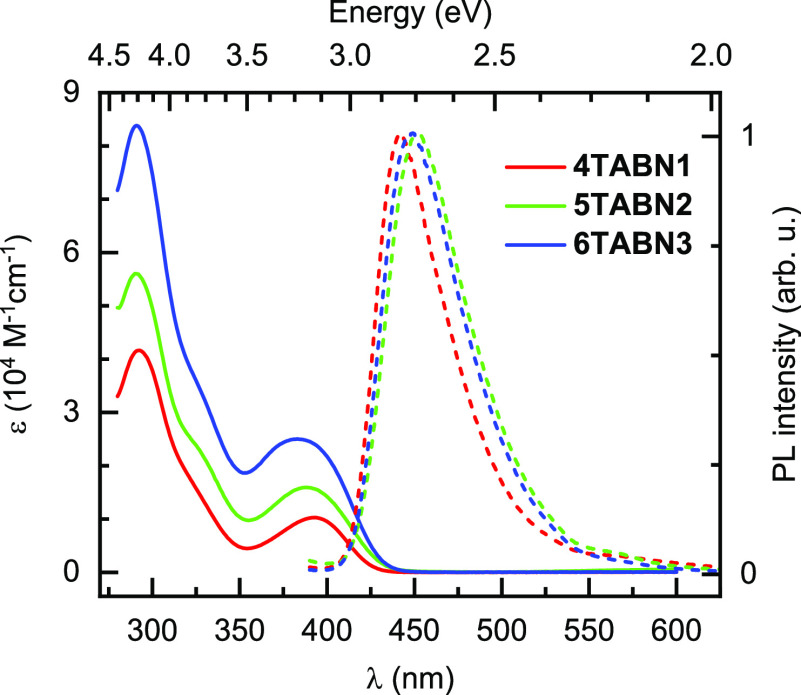
Absorption and steady-state emission spectra of **4TABN1**, **5TABN2**, and **6TABN3** in toluene at room temperature (λ_exc_ = 370 nm).

**Figure 4:**
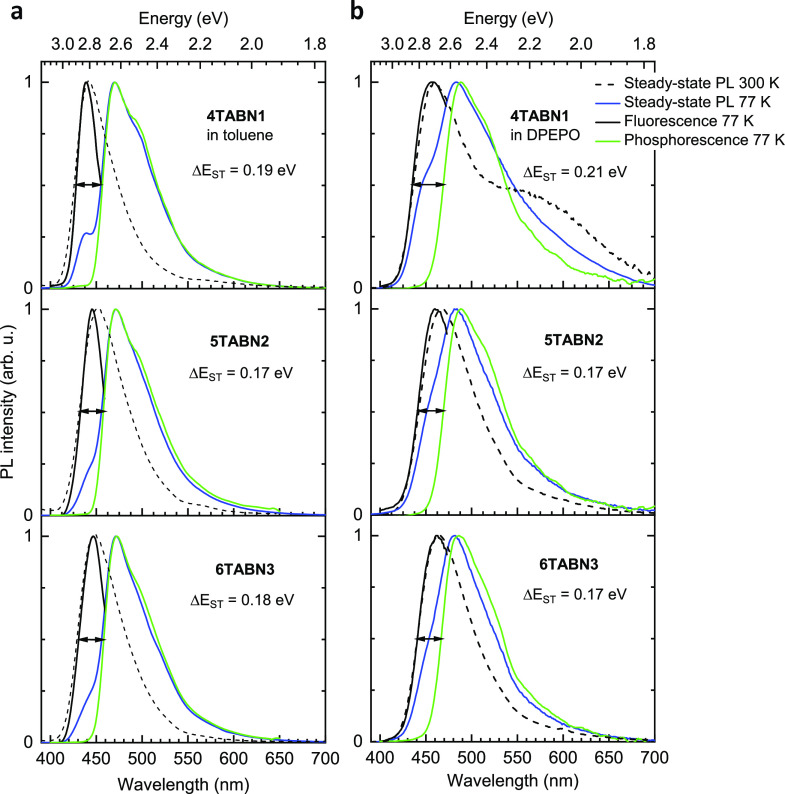
Steady-state emission (blue line), phosphorescence (delay 150 ms, gate 50 ms, green line), and fluorescence (black line), all at 77 K in (a) toluene solution and (b) 10 wt % film in DPEPO. “The black dashed line represents the steady-state emission at 300 K. λ_exc_ = 300 nm. The fluorescence was obtained as the difference between the steady-state emission and phosphorescence.

We started our photophysical study with low-concentration (ca. 10^–5^ M) toluene solution measurements to investigate the photophysical properties of the monomolecular species. Figure 3 presents the room temperature absorption and photoluminescence (PL) spectra of **4TABN1**, **5TABN2**, and **6TABN3**. All molecules show the same absorption profile but differ in the band intensity. Taking into account the nature of the donor and acceptor units, we assign the band at 4.23 eV (ca. 293 nm) to the π–π* absorption of the tolylamine donor unit,^38^ while absorption of the benzonitrile acceptor occurs at higher energy.^39^ The intensity of the band at 4.23 eV (293 nm) increases with increasing number of donors. There is also a broad, low-intensity, and structureless absorption band at 3.15 eV (394 nm) for **4TABN1** that is associated with intramolecular CT states (vide infra). The intensity of this band also increases with increasing number of D-A units (c.f. Table 1). A small blue shift of the maximum of the CT band (0.07 eV for **5TABN2** and 0.09 eV for **6TABN3**) implies on average a population of more twisted conformers. The Φ_PL_ values of the compounds in oxygen-free toluene are comparable at 18, 14, and 15% for **4TABN1**, **5TABN2**, and **6TABN3**, respectively. All three compounds show broad and structureless PL spectra that are characteristic of emission from CT states. The emission maxima vary narrowly from 2.71 to 2.76 eV (from 458 to 449 nm) as do the onset of the spectra, varying from 2.96 to 2.98 eV. For reference, the transient PL decays for the series in toluene are presented in Figure S29. For all three compounds, the prompt fluorescence has an identical decay of about 2.5 ns. The lifetime of the delayed fluorescence at room temperature is also very similar and about 100 ns, with a minor slow tail suggesting the additional presence of a small degree of triplet–triplet annihilation. We next cooled the solution to 77 K (Figure 4a) to ascertain the Δ*E*_ST_ gaps from the difference in energy from the onsets of the prompt fluorescence and phosphorescence spectra.

**Table 1:** Extinction Coefficients and Energetic Positions of the Photoluminescence Obtained from the Optical Spectroscopy of **4TABN1**, **5TABN2**, and **6TABN3** in Toluene and as 10 wt % DPEPO Films

	in toluene						in 10 wt % DPEPO film			
	λ_abs_^a^300 K	ε_CT_^b^(10^4^ M^–1^ cm^–1^)	λ_PL_^c^300 K	S_1_^d^(eV)	T_1_^d^(eV)	Δ*E*_ST_^e^(eV)	λ_PL_^c^300 K	S_1_^d^(eV)	T_1_^d^(eV)	Δ*E*_ST_^e^(eV)
**4TABN1**	3.16 eV (393 nm)	1.03	2.81 eV (441 nm)	2.95	2.76	0.19	2.67 eV (464 nm)	2.92	2.71	0.21
**5TABN2**	3.19 eV (389 nm)	1.59	2.76 eV (449 nm)	2.93	2.76	0.17	2.64 eV (470 nm)	2.89	2.72	0.17
**6TABN3**	3.24 eV (383 nm)	2.47	2.74 eV (452 nm)	2.95	2.77	0.18	2.66 eV (466 nm)	2.89	2.72	0.17

a Peak maximum of the CT absorption band.

b Molar extinction coefficient of the CT band.

c Peak maximum of the steady-state PL.

d S_1_ and T_1_ energies determined from the onset of the prompt fluorescence and phosphorescence spectra at 77 K, respectively.

e Δ*E*_ST_ = S_1_ – T_1_ with error of ±0.01.

Figure 4a shows the steady-state emission spectra in toluene glass at 77 K. The prompt fluorescence spectra in Figure 4a were extracted from the difference between the steady-state emission and phosphorescence spectra at 77 K. The prompt fluorescence is observed as a weak high-energy shoulder of the steady-state emission. Such behavior implies that at low temperature the main channel of deactivation of the singlet excited state is intersystem crossing (ISC) that leads to the population of the triplet state. When compared with the 300 K steady-state PL spectra, we note that there is no spectral shift of the prompt fluorescence that accompanies the change in temperature of the sample, which is typically observed for the emission from a CT state.^40^ Rather, this behavior is congruent with an emission from an LE state. The seemingly narrower linewidth is an artifact resulting from the normalization and subtraction procedure applied to obtain the prompt fluorescence spectra. We note too that prompt fluorescence is observed upon excitation of the low energy absorption band that we assigned to a CT state. However, the experimentally obtained extinction coefficient for this band is more typical for a state with a significant LE character.^41^ Based on the coexistence of features that are typical for CT and LE states, we assign the singlet transition to be of a mixed CT/LE character; this assignment is corroborated by the calculations (vide infra).

The phosphorescence spectra in toluene glass, recorded with a delay of 150 ms after excitation and a gate of 50 ms, are also shown in Figure 4a. The structured phosphorescence spectra of the three compounds all align, with a λ_PL_ of 2.62 eV (473 nm) at the peak. Since the origin of the phosphorescence comes from neither the donor nor the acceptor, it must originate from a new state. Based on the structured shape of the phosphorescence spectra as well as the insight gained from quantum chemical calculations (vide infra), we assign the lowest triplet state to be of mixed CT/LE character. As the phosphorescence spectra for the three compounds possess the same shape and energy, this reveals that each of the D-A units is not strongly coupled to one another. This conclusion is also supported by the analysis of their absorption spectra, where no new features appear along the series. The phosphorescence spectra occur at 0.17–0.19 eV lower in energy compared to their corresponding prompt fluorescence (Table 1), providing near identical Δ*E*_ST_ gaps. Compared to the reference **DCzBN1** (Δ*E*_ST_ = 0.31 eV in toluene),^42^ the ΔE_ST_ values for **4TABN1**, **5TABN2**, and **6TABN3** in toluene are much smaller, which should translate into compounds showing more pronounced TADF. For reference, a version of Figure 4 with an energy scale as the leading axis is shown in the ESI as Figure S30.

**Figure 5:**
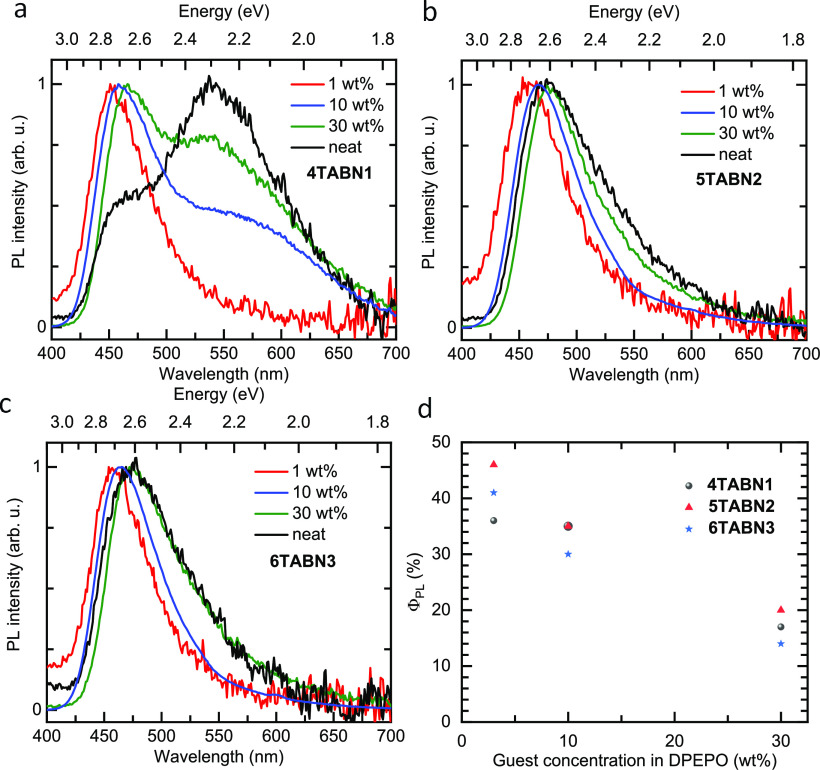
(a), (b), and (c) are the steady-state emission of the compounds in DPEPO thin films. (d) Φ_PL_ values at different doping concentrations of the films in DPEPO at RT. λ_exc_ = 315 nm.

With a view to assessing the potential of these compounds as emitters for OLEDs, we next evaluated their photophysical properties as 10 wt % blend films in DPEPO (bis[2-(diphenylphosphino)phenyl] ether oxide), which has a suitably high triplet energy of around 3.0 eV.^43^ The room temperature and 77 K PL spectra of the films are presented in Figure 4b. Similar to the solution-state PL spectra, all compounds show a broad and structureless high energy band, with λ_PL_ that are red-shifted by around 0.1 eV relative to their solution-state PL spectra (Table 1). The corresponding Commission Internationale to l’Éclairage (CIE) coordinates for **4TABN1**, **5TABN2**, and **6TABN3** are (0.16, 0.14), (0.16, 0.16), and (0.16, 0.16), respectively. The slightly lower energy of the S_1_ states of the DPEPO films than the corresponding values in solution, obtained using the onset of the prompt fluorescence spectra, can be attributed to the greater polarity of the DPEPO film than toluene.^44^ Similar to that observed in solution, the phosphorescence spectra are red-shifted, yielding Δ*E*_ST_ gaps ranging between 0.17 and 0.21 eV. In addition to the high energy band at around 2.65 eV, **4TABN1** shows a low energy band at 2.12 eV (585 nm), which was not observed in solution. For **5TABN2** and **6TABN3**, this low energy band is absent. To understand the origin of the low energy band in **4TABN1**, its steady-state emission was measured at a number of different concentrations (Figure 5).

Figure 5 shows the steady-state PL spectra of **4TABN1**, **5TABN2**, and **6TABN3** at 1 wt %, 10% wt %, and 30 wt % doped DPEPO films, as well as neat films. The observed red shift of the high energy band (around 2.7 eV) with increasing concentration for each of the emitters can be attributed to spectral diffusion.^45^ It is also evident that the significant low energy band (around 2.3 eV) present in the PL spectrum of **4TABN1** becomes more pronounced at higher doping concentrations in the film, which we assign to emission from an aggregate. To examine the nature of this aggregate, we measured the absorption spectrum of the neat film (Figure S31) and found it to be identical to that in toluene solution. Thus, there is either too low a concentration of the aggregate in the film to be discernible by absorption spectroscopy or the low energy PL band results from excimer/exciplex-like emission, which are known for their poor absorption cross sections.^46^ For **5TABN2**, and especially **6TABN3**, this low energy feature in the PL spectrum is strongly suppressed (Figure 5b,c). The Φ_PL_ values at 3, 10, and 30 wt % doping are summarized in Figure 5d. Although the low energy band is no longer visible in **5TABN2** and **6TABN3**, the decrease in Φ_PL_ is similar for all compounds, suggesting that there is still some intermolecular interaction of the latter two compounds at higher doping concentrations in the DPEPO films.

**Figure 6:**
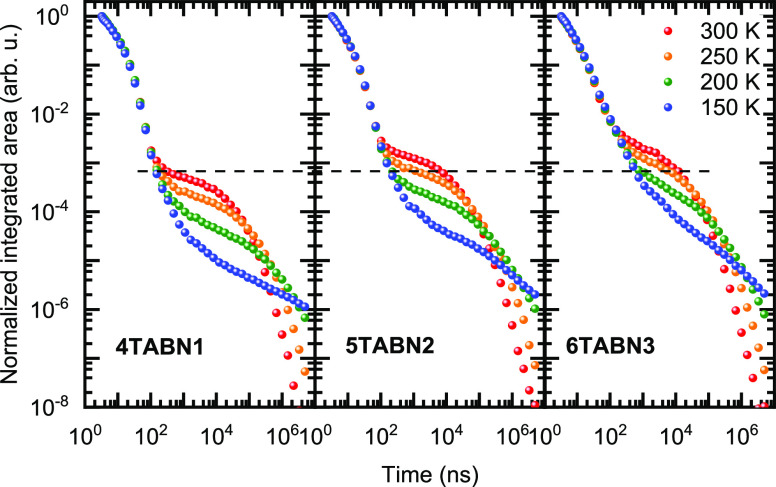
Transient photoluminescence decay of the blend films in 3 wt % DPEPO films (λ_exc_ = 355 nm). Integrated across the full spectral range.

We next evaluated the evolution of the time-resolved PL decays of the films as a function of temperature (Figure 6).

Each decay consists of two regimes: the prompt fluorescence, lasting up until around 100 ns, and the delayed fluorescence, on the order of microseconds. The prompt fluorescence is insensitive to temperature, but the intensity of the delayed fluorescence is temperature dependent. Comparing the delayed fluorescence amplitudes, it is evident that the delayed fluorescence is more pronounced in **5TABN2** and **6TABN3**, which is consistent with a slightly smaller Δ*E*_ST_ in these two compounds (Table 1). The photophysical parameters extracted from the analysis of the transient PL decay curves according to the methods of Baleizão and Berberan-Santos and Dias et al*.*^21,22^ are summarized in Table 2. The activation energies, extracted from an Arrhenius analysis of the temperature-dependent time-resolved PL decays, are in the range between 70 and 80 meV (Figure S32), i.e., less than half of Δ*E*_ST_. We tentatively ascribe the difference between the estimated Δ*E*_ST_ and the activation energies to the presence of higher lying triplet states that facilitate RISC.^47^

**Table 2:** Efficiencies and Rate Constants of **4TABN1**, **5TABN2**, and **6TABN3** Films in DPEPO (3 wt %)

	Φ_PL_^a^(%)	Φ_DF_/Φ_PL_^b^	τ_PF_^c^(ns)	τ_DF_^d^ (μs)	*k*_r_^S^^e^ (×10^7^, s^–1^)	*k*_nr_^S^^f^ (×10^7^, s^–1^)	Φ_ISC_^g^	*k*_ISC_^h^ (×10^8^, s^–1^)	*k*_RISC_^i^ (×10^4^, s^–1^)
**4TABN1**	36	2.8	11.0	113	0.8	1.7	0.75	0.7	3.5
**5TABN2**	46	4.1	7.0	92	1.3	1.5	0.8	1.2	5.6
**6TABN3**	41	4.4	7.0	67	1.2	1.6	0.8	1.2	7.7

a Total Φ_PL_ (λ_exc_ = 315 nm).

b Ratio of the integrated areas of delayed and prompt emission.

c Lifetime of the prompt fluorescence estimated using a mono-exponential fit at 300 K.

d Average lifetime of delayed emission at 300 K.

e Radiative decay rate constant of singlet excitons.

f Non-radiative decay rate constant of singlet excitons excluding *k*_ISC_.

g Intersystem crossing yield.

h Intersystem crossing rate constant.

i Reverse intersystem crossing rate constant.

Starting with the singlet-state properties, we note that the radiative decay rate constants do not evolve in parallel with the molar extinction coefficients (Figure 3, Table 1); more specifically, the latter evolve linearly with the number of repeating units, as expected. Indeed, if we divide the extinction coefficients presented in Figure 3 by 1.5, 2.5, and 3.5, numbers that represent the amount of “repetition” in the oligomers (where an integer number stands for a full D-A pair while 0.5 represents the remaining part of the molecule, Figure S33), a common value of 0.7 × 10^4^ M^–1^ cm^–1^ per D-A repeat unit is obtained. For molecules behaving as single chromophore species, the radiative decay rate constant is expected to scale with the extinction coefficient. Therefore, the ratio in ε_**CT**_ values between **5TABN2** and **4TABN2** of 1.67 translates into an expected radiative decay rate in **5TABN2** of 1.67 × 0.80 × 10^7^ s^–1^ = 1.35 × 10^7^ s^–1^, which is exactly the measured value in Table 2. However, there is no further increase in the radiative decay rate going from **5TABN2** to **6TABN3** showing that, in the emissive state, the wavefunction is confined over on average 2.5 repeating units. This localization of the excited-state wavefunction, i.e., the shorter conjugation length after geometric relaxation in the excited state, is likely induced by more strongly distorted conformations in the relaxed excited state, which also results in neither S_1_ nor T_1_ showing an appreciable red shift along the series (Table 1). The larger *k*_RISC_ cannot be due to the difference in Δ*E*_ST_ because the measured activation energy for TADF is the same for all three compounds (c.f. Figure S32).

From the photophysical investigation, we have demonstrated that the PL emission is largely similar for each of the three compounds, with the presence of the additional low energy band in **4TABN1** that we ascribe to an excimer or exciplex-like species.

**Figure 7:**
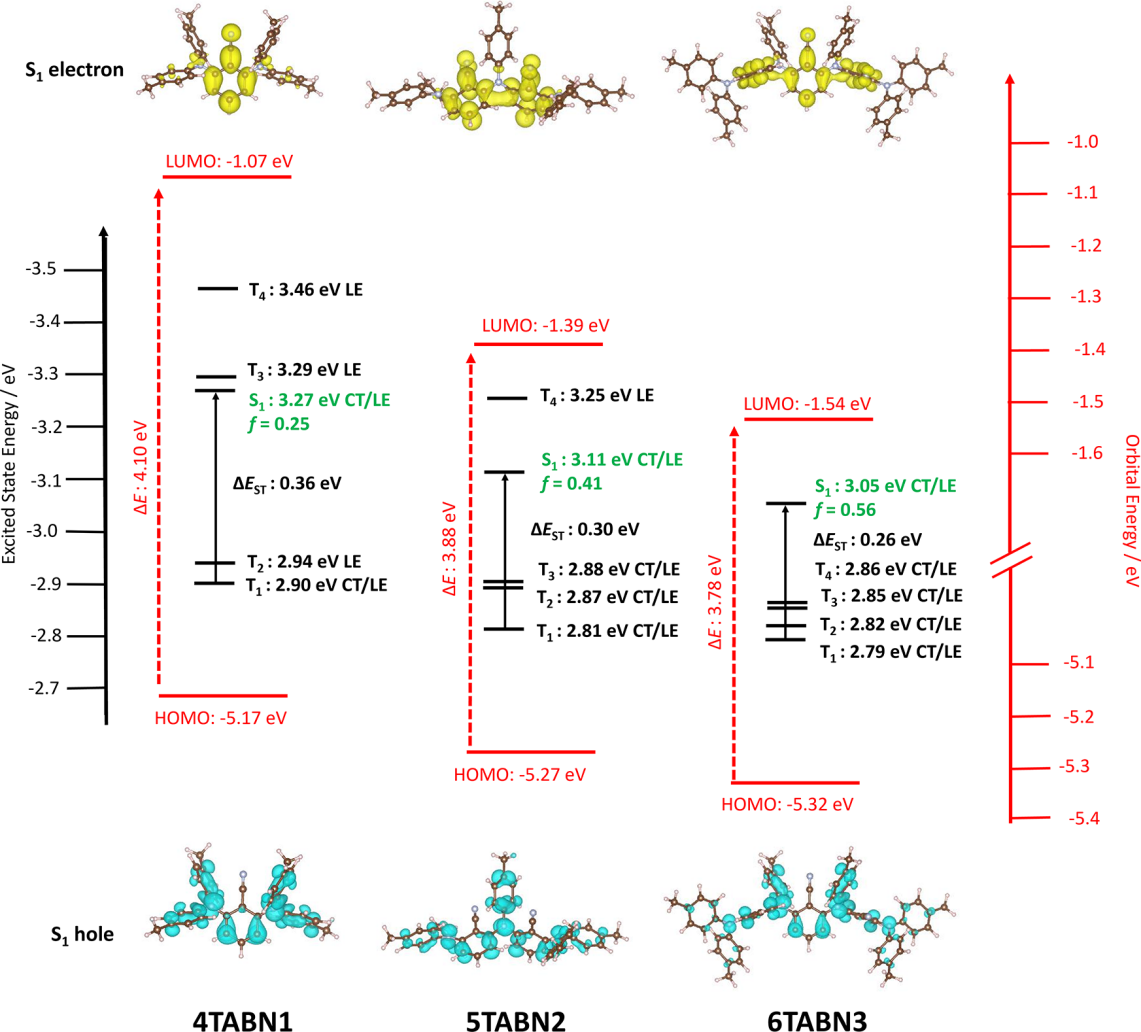
Right axis in red: HOMO/LUMO energies calculated for the ground-state geometry. Left axis in black: vertical transition energies for S_1_, T_1_, T_2_, T_3_, and T_4_ and their assigned natures. Also shown are the hole (blue) and electron (yellow) densities of the S_1_ state. Calculated at the PBE0/6-31G(d,p) level, where *f* is the oscillator strength. Isovalue = 0.001.

We finally employed DFT calculations and time-dependent DFT calculations within the Tamm–Dancoff approximation (TDA-DFT) using the PBE0 functional with the 6-31G(d,p) basis set to model the optoelectronic properties of the three emitters (Figure 7, Tables S3 and S4). This protocol has been widely applied by us and others to accurately model the optoelectronic properties of TADF emitters.^48,49^ In expanding the structure from one benzonitrile acceptor to two and three acceptor units, there is a progressive stabilization of the HOMO energy from −5.17 eV in **4TABN1** to −5.27 eV in **5TABN2** and −5.32 eV in **6TABN3**. The same trend is observed for the LUMO energies where a more pronounced stabilization from −1.07 eV in **4TABN1** to −1.39 eV in **5TABN2** and −1.54 eV in **6TABN3** is observed. All energies are derived from the ground-state geometries. The corresponding HOMO–LUMO gap, Δ*E*, decreases from **4TABN1** to **6TABN3**, in corroboration with the experimental HOMO/LUMO values (vide supra).

In line with decreasing Δ*E*, there is stabilization of the S_1_ state from 3.27 to 3.11 and 3.05 eV for **4TABN1**, **5TABN2**, and **6TABN3**, respectively. This decreasing S_1_ energy is not reflected in the experimentally obtained absorption spectra (calculations that incorporate a toluene solvent corroborate those conducted in the gas phase as shown in Table S5). We ascribe the discrepancies due to the large conformational degrees of freedom in this series, especially in the larger molecules, while the calculations focus only on a fixed geometry. Across the series, an increase in oscillator strength is apparent from 0.25, 0.41, and 0.56 for **4TABN1**, **5TABN2**, and **6TABN3**, respectively, and is expected owing to increasing conjugation in the ground-state geometry with increasing size. This is in line with the increasing experimental ε values observed in toluene (vide supra) and are comparable to oscillator strengths in toluene (Table S4). Regarding the properties of the intermediate triplet states, the higher-lying triplet states (T_3_–T_4_) are stabilized more significantly compared to S_1_ along the series (Table S3). For example, the energy of T_3_ changes from 3.29 to 2.88 and 2.85 eV along the series, with T_3_ being more than 200 meV below S_1_ for **5TABN2** and **6TABN3**, respectively. Given such a large and unambiguous energetic difference between S_1_ and T_3_, we nevertheless expect the predicted higher-lying triplet states to play a role as intermediate triplet states in **5TABN2** and **6TABN3**, even when the DFT calculations predict greater conjugation in the oligomers than experimentally observed. The calculations show that the number of intermediate triplet states increase along the series (Figure 7), with one, two, and three intermediate triplet states present in **4TABN1**, **5TABN2**, and **6TABN3**, respectively. Their presence would support the trend in experimentally determined *k*_ISC_. However, having intermediate triplet states alone is not sufficient to explain the enhanced TADF in **5TABN2** and **6TABN3**. An efficient SOC between the singlet and triplet manifolds is enabled by states of different symmetry and electronic configuration.^7,8^ This means that a strong SOC can be enabled if the transition between singlet and triplet states involves a change of molecular orbital type. We therefore quantified the degree of CT versus LE character of the excited states using the ϕ_S_ metric,^50^ wherein we assign an excited state with ϕ_S_ = 1 as being LE, ϕ_S_ = 0 as CT, and intermediate values as mixed LE/CT states along with the predicted SOC values. For each of the emitters, both the T_1_ and S_1_ states are predicted to be of mixed CT/LE character (ϕ_S_ = 0.5–0.6, see Table S3 and Figures S35–S37), consistent with the photophysical investigation. For **4TABN1**, the higher-lying triplet states, T_2_–T_4_, are all LE with ϕ_S_ > 0.75, while for **5TABN2** and **6TABN3**, the intermediate triplet states are of mixed LE/CT character. The analysis of the nature of the excited states indeed shows that intermediate triplet states possess a significant LE character (ϕ_S_ = 0.7 for T_2_ and T_3_, see Table S3 and Figures S35–S37), different to that of S_1_, which is more CT in nature. Calculated SOC matrix elements between S_1_ and the T_1_–T_3_ states (Table S5) all show promisingly large values at above 1.0 cm^–1^ for LE triplet states and between 0.13 and 0.74 cm^–1^ for the CT/LE triplet states, indicating possible routes for RISC involving these states. The Δ*E*_ST_ gaps decrease from 0.37 to 0.30 and 0.26 eV to Δ*E*_ST_ values of 0.33, 0.23, and 0.19 eV if the energy difference between S_1_ and the highest intermediate triplet states are considered, for **4TABN1**, **5TABN2**, and **6TABN3**, respectively. The small singlet–triplet gaps predicted for **5TABN2** and **6TABN3** support the experimentally determined increasing the *k*_RISC_ rate along the series. Further detailed discussion regarding the computations are found in the Supporting information.

## Conclusions

We present a novel approach to increase the number of quasi-degenerate states, and by consequence the rate for reverse intersystem crossing, by increasing the number of repeat units in a highly twisted D-A oligomer. In contrast to earlier related work, we are able to preserve the emission energy and spectral shape.^51,14^ This was achieved by using an *ortho* substitution pattern of the donor units about the BN acceptor that also translated to a highly twisted conformation for each of the emitters. This molecular design results in only limited conjugation throughout the oligomer so that the blue emission is preserved along the series, and it also limits the interaction between hole and electron densities, resulting in each of the emitters retaining their small Δ*E*_ST_. In this way, our approach provides a route toward efficient blue TADF emission.

## Data Availability

The research data supporting this publication can be accessed at https://doi.org/10.17630/e05eb3f8-b881-4f5a-b09e-3412f754ecff.

## Supporting Information Available

^1^H NMR and ^13^C NMR spectra, GCMS, HRMS, EA and HPLC; detailed photophysical methods and spectra; supplementary computational data and coordinates; crystallographic data (PDF)
**4TABN1** (CCDC 2218300) and **5TABN**2 (CCDC 2218301) (TXT)
