# Supplementary material for: An Oligomer Approach for Blue Thermally Activated Delayed Fluorescent Emitters Based on Twisted Donor–Acceptor Units
Source: Chem Mater. 2023 Feb 28;35(5):2027–37. doi: 10.1021/acs.chemmater.2c03438 (PMC10018739; doi:10.1021/acs.chemmater.2c03438)
Supplement: Supplementary file 1 — cm2c03438_si_001.pdf [file cm2c03438_si_001.pdf]

SUPPORTING INFORMATION

An oligomer approach for blue thermally activated delayed fluorescent emitters based on twisted donor-acceptor units

Eimantas Duda,[#a] Subeesh Madayanad Suresh,[b#] David Hall,[b,c] Sergey Bagnich,[a] Rishabh Saxena,[a]

David B. Cordes,[b] Alexandra M. Z. Slawin,[b] David Beljonne,[c] Yoann Olivier,[c,d] Anna Köhler[a]**

*and Eli Zysman-Colman[b]**

[a]Soft Matter Optoelectronics, BIMF & BPI, University of Bayreuth, Universitätsstraße 30, 95447 Bayreuth, Germany. E-mail: anna.koehler@uni-bayreuth.de

[b]Organic Semiconductor Centre, EaStCHEM School of Chemistry, University of St Andrews, St Andrews, UK, KY16 9ST. E-mail: eli.zysman-colman@st-andrews.ac.uk

[c]Laboratory for Chemistry of Novel Materials, University of Mons, 7000, Mons, Belgium.

[d] Unité de Chimie Physique Théorique et Structurale & Laboratoire de Physique du Solide, Namur Institute of Structured Matter, Université de Namur, Rue de Bruxelles, 61, 5000 Namur, Belgium.

[#] Contributed equally

Table of Contents

	Pages
Synthesis	S2
1H, 13C NMR spectra and HRMS	S8
Electrochemistry	S21
Photophysical Characterization	S23
Supplementary Calculations	S26
Supplementary XRD data	S37
References	S38

Synthesis

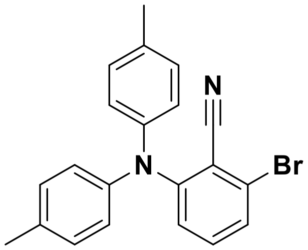

2-bromo-6-(di-*p*-tolylamino)benzonitrile (1)

A Schlenk tube was held under nitrogen and charged with di-*p*-tolylamine (3.55 g, 18.0 mmol, 1 equiv.) and Cs_2CO_3 (17.6 g, 54.0 mmol, 3 equiv.). Solvent (DMF, 15 mL) was added and stirred for 10 min at RT. To this solution, 2-bromo-6-fluorobenzonitrile (3.60 g, 18.0 mmol, 1 equiv.) was added and the reaction mixture heated to 140 °C for 12 h. The reaction was cooled to room temperature and diluted with ethyl acetate (200 mL). The organic layer was washed with water (3 × 100 mL) and then dried on anhydrous sodium sulfate. The solvent was removed under reduced pressure. The crude product was purified by flash chromatography on silica gel (10 : 90 EtOAc : *n*-hexane). The corresponding fractions were combined and concentrated under reduced pressure. The product was precipitated by adding cold methanol (20 mL), which was filtered to afford the desired product as a yellow crystalline solid. **Yield:** 68% (4.60 g). **R_f**: **0.46** (10 : 90 EtOAc : *n*-hexane on silica gel). **Mp**: 147-150 °C. **^{1}H NMR (400 MHz, Aetone-*d*6) δ (ppm)**: 7.51-7.48 (m, 2 H), 7.17-7.12 (m, 5 H), 6.93- 6.88 (m, 4 H), 2.30 (s, 6 H). **^{13}C NMR (100 MHz, Aetone-*d*6) δ (ppm)**: 153.26, 144.88, 134.53, 133.50, 130.06, 127.59, 126.25, 126.11, 124.42, 124.01, 115.03, 112.31, 19.94. **GC-MS [M]$^+$** Calculated: 376.06 ($C_{21}H_{17}BrN_2$); Found: 376.05.

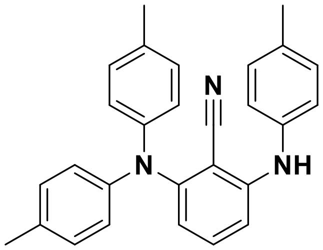

2-(di-*p*-tolylamino)-6-(*p*-tolylamino)benzonitrile (2)

To compound **1** (3 g, 8.0 mmol, 1 equiv.) under a nitrogen atmosphere were added *p*-toluidine (0.85 g, 8.0 mmol, 1 equiv.), $Pd_2(dba)_3$ (0.18 g, 0.2 mmol, 0.025 equiv.), SPhos (0.16 g, 0.40 mmol, 0.05 equiv.) and sodium *tert*-butoxide (3.0 g, 31 mmol, 3.9 equiv.). Solvent (toluene, 15 mL) was then added and the reaction mixture heated to 110 °C for 12 h. The reaction was cooled to room temperature and diluted with ethyl acetate (150 mL). The organic layer was washed with water (3 × 100 mL) and then dried with anhydrous sodium sulfate. The solvents were removed under reduced pressure. The crude product was purified by chromatography on silica gel (20 : 80 EtOAc : *n*-hexane). The corresponding fractions were combined and concentrated under reduced pressure to afford a yellow solid, which was then filtered and washed with pentane. **Yield:** 61% (1.95 g). **R_f**: 0.51 (10 : 90 EtOAc : hexanes on silica gel). **Mp**: 173-176 °C. **^{1}H NMR (400 MHz, Acetone-*d6*) δ (ppm)**: 7.34 (td, *J* = 8.3, 0.6 Hz, 1H), 7.25 (s, 1H), 7.19 (s, 1H), 7.13 – 7.08 (m, 4H), 6.95 – 6.84 (m, 5H), 6.52 (dd, *J* = 8.0, 0.9 Hz, 1H), 2.31 (s, 3H), 2.29 (s, 6H). **^{13}C NMR (100 MHz, Acetone-*d6*) δ (ppm)**: 152.02, 150.05, 145.28, 138.41, 133.97, 133.01, 132.41, 129.83, 129.77, 123.44, 121.96, 117.63, 115.23, 109.84, 97.79, 19.95, 19.90. **GC-MS [M]$^+$** Calculated: 403.20 ($C_{28}H_{25}N_3$); Found: 403.20.

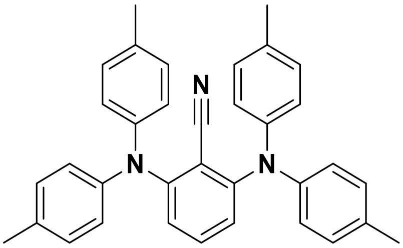

2,6-bis(di-*p*-tolylamino)benzonitrile (4TABN1)

Using a similar protocol to that of compound **2**, **4TABN1** was obtained from compound **1** by replacing *p*-toluidine with di-*p*-tolylamine. The crude product was purified by chromatography on silica gel (10 : 90 EtOAc : hexanes). The corresponding fractions were combined and concentrated under reduced pressure to afford a yellow oil, which was sonicated in pentane. The precipitate formed was filtered, re-dissolved in minimum amount of DCM and precipitated by adding cold pentane. The pure product obtained was filtered and washed with pentane to afford a pale yellow solid. **Yield:** 83% (3.32 g). **R_f**: 0.46 (10 : 90 EtOAc : hexane on silica gel). **Mp**: 176-178 °C. **^{1}H NMR (500 MHz, $CDCl_3$) δ (ppm)**: 7.22 (t,

J = 8.2 Hz, 1H), 7.03 (d, J = 8.3 Hz, 7H), 6.90 (d, J = 8.4 Hz, 6H), 6.73 (d, J = 8.2Hz, 2 H), 2.29 (s, 11H). **^{13}C NMR (125 MHz, $CDCl_3$) δ (ppm)**: 152.98, 144.87, 133.30, 132.84, 129.70, 123.73, 120.98, 115.07, 106.39, 20.73. **GC-MS [M]$^+$** Calculated: 493.21 ($C_{35}H_{31}N_3$); Found: 493.55. 98% pure on HPLC trace analysis. Anal. Calcd. for $C_{35}H_{31}N_3$: C, 85.16%; H, 6.33%; N, 8.51%. Found: C 85.17%; H, 6.14%; N, 8.39%.

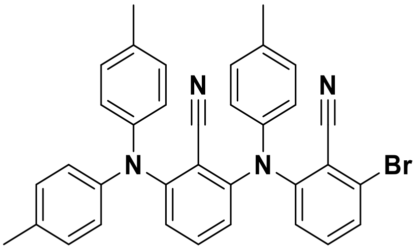

2-bromo-6-((2-cyano-3-(di-*p*-tolylamino)phenyl)(*p*-tolyl)amino)benzonitrile (3)

A Schlenk tube was held under nitrogen and charged with compound **2** (1.70 g, 4.21 mmol, 1 equiv.) and Cs_2CO_3 (4.12 g, 12.6 mmol, 3 equiv.). Solvent (DMF, 12 mL) was added and stirred for 10 min at RT. To this solution was added 2-bromo-6-fluorobenzonitrile (1.26 g, 6.32 mmol, 1.5 equiv.) and the reaction mixture was heated to 140 °C for 12 h. The reaction was cooled to room temperature and diluted with ethyl acetate (200 mL). The organic layer was washed with water (3 × 100 mL) and then dried over anhydrous sodium sulfate. The solvent was removed under reduced pressure. The crude product was purified by flash chromatography on silica gel (20 : 80 EtOAc : hexane). The corresponding fractions were combined and concentrated under reduced pressure to get a yellow oil, which was sonicated in pentane. The precipitated product was filtered and washed with pentane to afford the desired product as a yellow solid. **Yield:** 89 % (2.18 g). **R$_f$**: 0.48 (20 : 80 EtOAc : hexane on silica gel). **Mp**: 195-196 °C. **^{1}H NMR (500 MHz, DMSO-d6) δ (ppm)**: 7.59 – 7.44 (m, 3H), 7.21-7.14 (m, 2H), 7.12 – 7.03 (m, 5H), 7.02 – 6.93 (m, 2H), 6.89 – 6.82 (m, 5H), 6.78 (dd, J = 8.2, 0.9 Hz, 1H), 2.30 (s, 3H), 2.25 (s, 6H). **^{13}C NMR (125 MHz, DMSO-d6) δ (ppm)**: 152.74, 152.45, 151.64, 144.93, 143.89, 135.65, 135.46, 134.90, 132.99, 132.92, 130.81, 130.41, 128.55, 126.82, 125.35, 124.82, 123.70, 123.64, 122.09, 115.65, 114.88, 110.74, 106.93, 20.95, 20.86. **GC-MS [M]$^+$** Calculated: 582.14 ($C_{35}H_{27}BrN_4$); Found: 582.10.

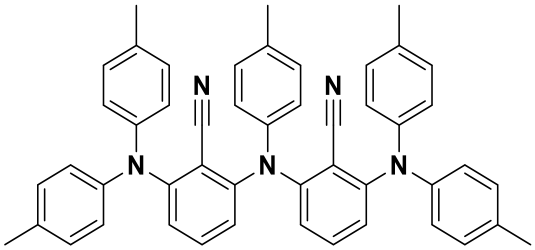

6,6'-(p-tolylazanediyl)bis(2-(di-p-tolylamino)benzonitrile) (**5TABN2**)

5TABN2 was obtained following the same protocol used for **4TABN1** starting from compound **3**. Purified by silica gel column chromatography (20 : 80 EtOAc : hexane). Pale yellow solid. **Yield**: 53% (2.97 g). **R*f***: 0.55 (20 : 80 EtOAc : hexane on silica gel). **Mp**: 169-173 °C. **^{1}H NMR (500 MHz, DMSO-d6) δ (ppm):** 7.47 (t, *J* = 8.2 Hz, 2H), 7.12 (dd, *J* = 8.6, 0.8 Hz, 2H), 7.08 – 7.00 (m, 8H), 6.91 – 6.84 (m, 2H), 6.83 – 6.73 (m, 12H), 2.26 (s, 3H), 2.22 (s, 12H). **^{13}C NMR (125 MHz, DMSO-d6) δ (ppm):** 152.28, 151.79, 144.69, 143.97, 134.87, 133.86, 132.36, 130.28, 130.07, 124.04, 123.27, 123.12, 121.49, 114.68, 106.79, 20.64, 20.56. **HRMS [M+1]$^+$** Calculated: 700.34 ($C_{49}H_{41}N_5$); Found: 700.3404. 98% pure on HPLC trace analysis. Anal. Calcd. for $C_{49}H_{41}N_5$: C, 84.09%; H, 5.90%; N, 10.01%. Found: C 84.15%; H, 5.76%; N, 9.81%.

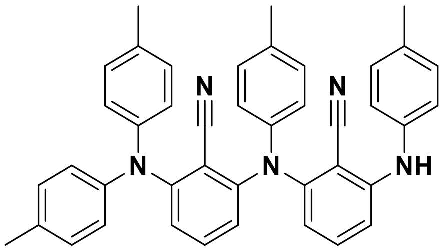

2-((2-cyano-3-(*p*-tolylamino)phenyl)(*p*-tolyl)amino)-6-(di-*p*-tolylamino)benzonitrile (**4**)

To compound **3** (4.0 g, 6.9 mmol, 1 equiv.) under a nitrogen atmosphere were added *p*-toluidine (0.73 g, 6.9 mmol, 1 equiv.), $Pd_2(dba)_3$ (0.19 g, 0.21 mmol, 0.03 equiv.), SPhos (0.17 g, 0.41 mmol, 0.06 equiv.) and sodium *tert*-butoxide (2.0 g, 21 mmol, 3 equiv.). Solvent (toluene, 20 mL) was then added and the reaction mixture heated to 110 °C for 5 h. The reaction was cooled to room temperature and diluted with ethyl acetate (250 mL). The organic layer was washed with water (3 × 150 mL) and then dried over anhydrous sodium sulfate. The solvents were removed under reduced pressure. The crude

product was purified by chromatography on silica gel (10 : 90 EtOAc : hexanes). The corresponding fractions were combined and concentrated under reduced pressure to afford a yellow fluffy solid, which was then filtered and washed with hexane. **Yield**: 63% (2.65 g). **R*f***: 0.19 (10 : 90 EtOAc : hexane on silica gel). **Mp**: 193-196 °C. **^{1}H NMR (500 MHz, DMSO-d6) δ (ppm)**: 8.17 (s, 1H), 7.51 (t, *J* = 8.2 Hz, 1H), 7.31 (t, *J* = 8.3 Hz, 1H), 7.20 – 6.97 (m, 10H), 6.92 – 6.78 (m, 8H), 6.74 (dd, *J* = 8.2, 0.9 Hz, 1H), 6.40 (dd, *J* = 8.0, 0.9 Hz, 1H), 2.27 (s, 3H), 2.25 (s, 3H), 2.24 (s, 6H). **^{13}C NMR (125 MHz, DMSO-d6) δ (ppm):** 152.52, 152.19, 151.79, 150.20, 145.00, 144.47, 139.23, 135.22, 134.75, 133.83, 132.66, 132.07, 130.50, 130.37, 130.11, 124.23, 123.50, 123.40, 121.93, 121.18, 116.76, 116.05, 114.93, 111.98, 107.93, 97.42, 20.92, 20.89, 20.86. **HRMS [M+H]$^{+}$** Calculated: 610.2892 ($C_{42}H_{35}N_5$); Found:610.2960.

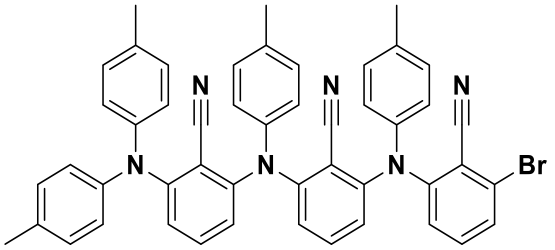

2-bromo-6-((2-cyano-3-((2-cyano-3-(di-*p*-tolylamino)phenyl)(*p*-tolyl)amino)phenyl)(*p*-tolyl)amino)benzonitrile (**5**)

Following the procedure for compound **3**, the target compound **5** was obtained from **4.** Purified by silica gel column chromatography (20 : 80 EtOAc : hexane). Yellow solid. **Yield:** 81 % (2.69 g). **R*f***: 0.35 (20 : 80 EtOAc : hexanes on silica gel). **Mp**: 182-185 °C. **^{1}H NMR (500 MHz, DMSO-d6) δ (ppm)**: 7.53-7.51 (m, 2H), 7.46 (td, J = 8.2, 1.7 Hz, 2H), 7.13 (dd, J = 10.6, 8.3 Hz, 4H), 7.06 (d, J = 8.2 Hz, 4H), 7.01 (dd, J = 8.2, 1.1 Hz, 1H), 6.90 – 6.77 (m, 12H), 2.26 (2 singlets, 6H), 2.23 (s, 6H). **^{13}C NMR (125 MHz, DMSO-d6) δ (ppm)**: 153.17, 152.37, 152.30, 151.88, 151.70, 144.97, 143.91, 143.56, 135.33, 135.06, 133.93, 133.84, 133.68, 132.95, 130.46, 130.29, 129.91, 127.75, 127.02, 125.27, 125.13, 124.88, 123.93, 123.89, 122.66, 122.09, 121.99, 121.30, 115.32, 114.93, 114.25, 110.80, 105.55, 105.00, 21.03, 21.02, 20.90. **HRMS [M]$^{+}$** Calculated: 788.2263 ($C_{42}H_{37}BrN_6$); Found:788.2261.

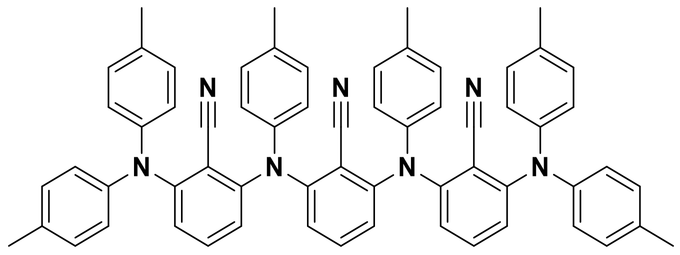

6,6'-((2-cyano-1,3-phenylene)bis(p-tolylazanediyl))bis(2-(di-p-tolylamino)benzonitrile) (**6TABN3**)

6TABN3 was obtained following the same protocol for **4TABN1** starting from compound **5**. The crude product was purified by chromatography on silica gel (10 : 90 EtOAc : pentane). The corresponding fractions were combined and concentrated under reduced pressure, methanol (20 mL) was added and placed in a freezer (-20 °C) for 30 min. The precipitate formed was filtered. Yellow solid. **Yield:** 38% (2.74 g). **R*f***: 0.13 (10 : 90 EtOAc : hexanes on silica gel). **Mp**: 169-173 °C. **^{1}H NMR (500 MHz, DMSO-d6) δ (ppm):** 7.49-7.43 (m, 3H), 7.12 (d, *J* = 8.1 Hz, 4H), 7.06 (d, *J* = 8.4 Hz, 8H), 6.87 (d, *J* = 8.3 Hz, 4H), 6.83 – 6.73 (m, 14H), 2.26 (s, 6H), 2.23 (s, 12H). **^{13}C NMR (125 MHz, DMSO-d6) δ (ppm):** 152.55, 152.19, 151.89, 145.01, 144.30, 135.17, 135.01, 134.23, 132.62, 130.58, 130.37, 124.39, 123.53, 123.40, 122.35, 121.81, 115.01, 114.70, 107.01, 105.64, 20.94, 20.85. **HRMS [M+1]$^+$** Calculated: 906.42 ($C_{63}H_{51}N_7$); Found:906.4236. 96% pure on HPLC trace analysis. Anal. Calcd. for $C_{63}H_{51}N_7$: C, 83.51%; H, 5.67%; N, 10.82%. Found: C 84.31%; H, 5.64%; N, 10.60%.

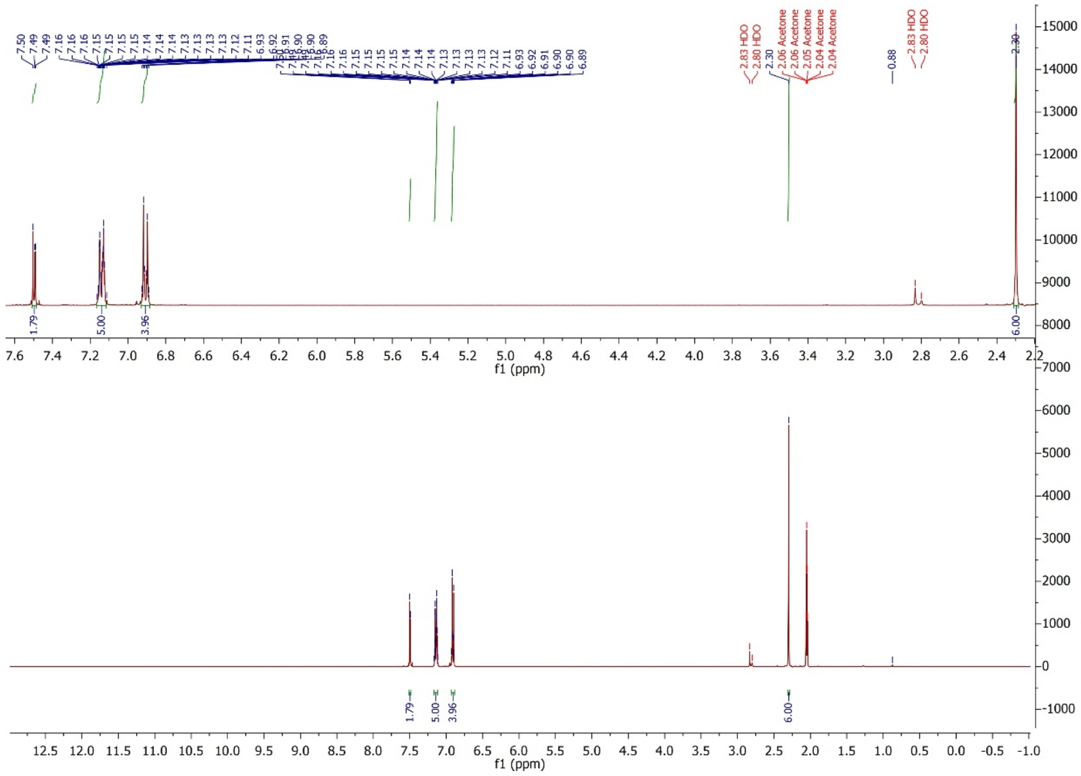

Figure **S1**. ^{1}H NMR of **1** in Acetone-d_6.

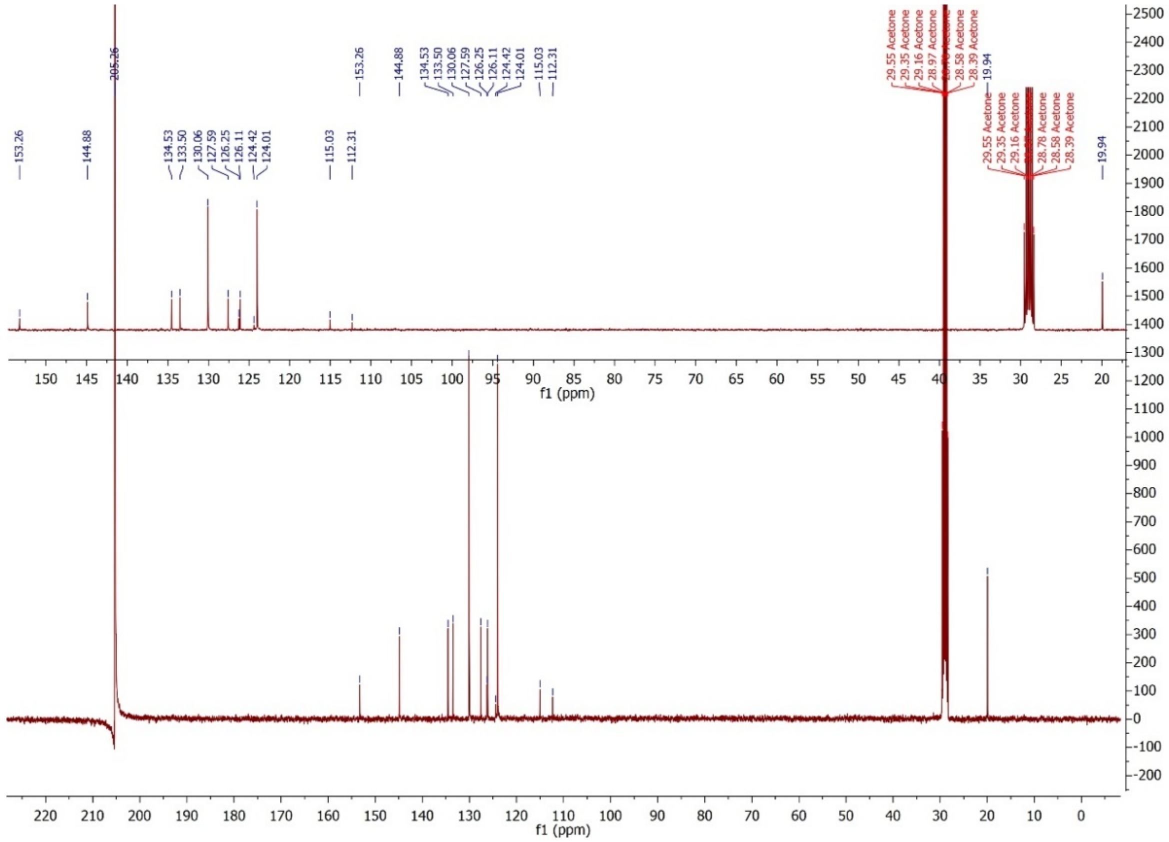

Figure **S2**. ^{13}C NMR of **1** in Acetone-d_6.

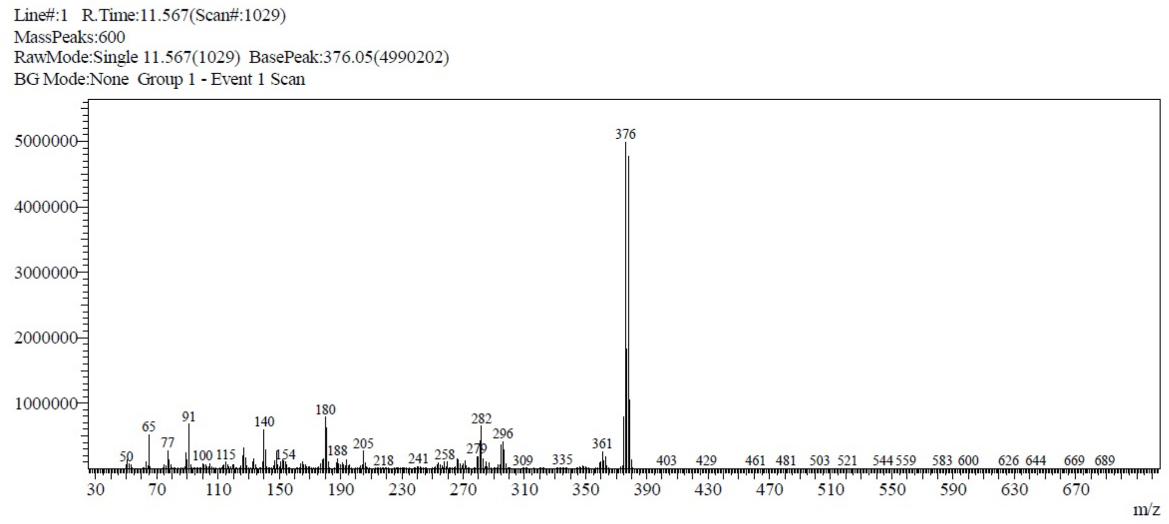

Figure **S3**. GCMS of **1**.

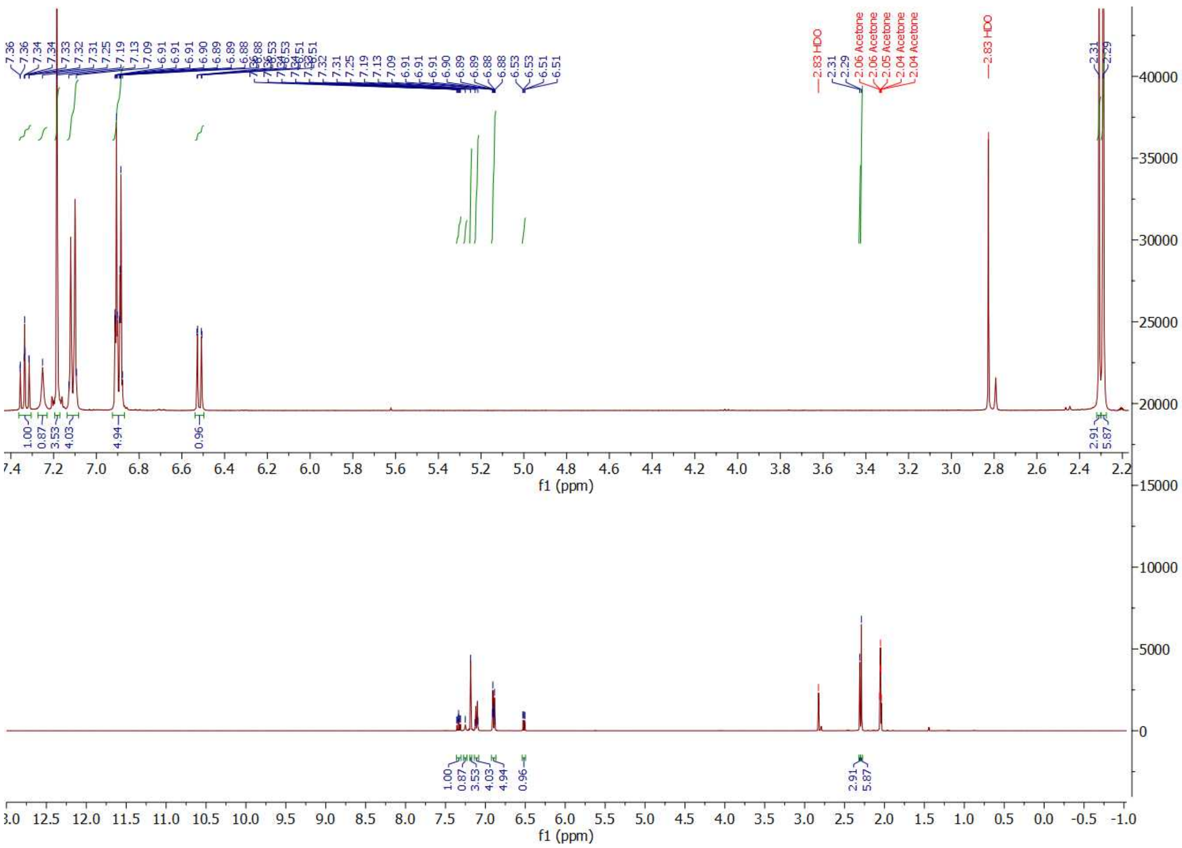

Figure **S4**. ^{1}H NMR of **2** in Acetone-d_6.

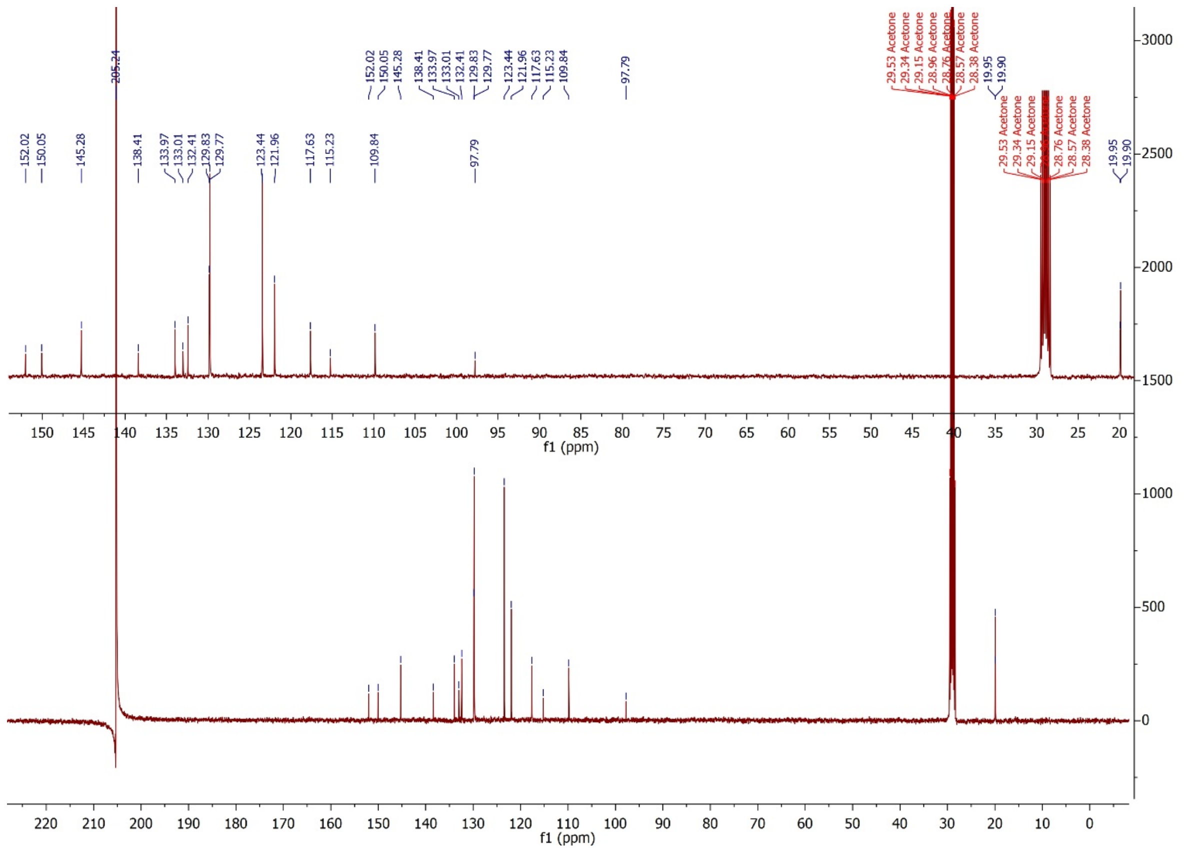

Figure **S5**. ^{13}C NMR of **2** in Acetone-d$_6$.

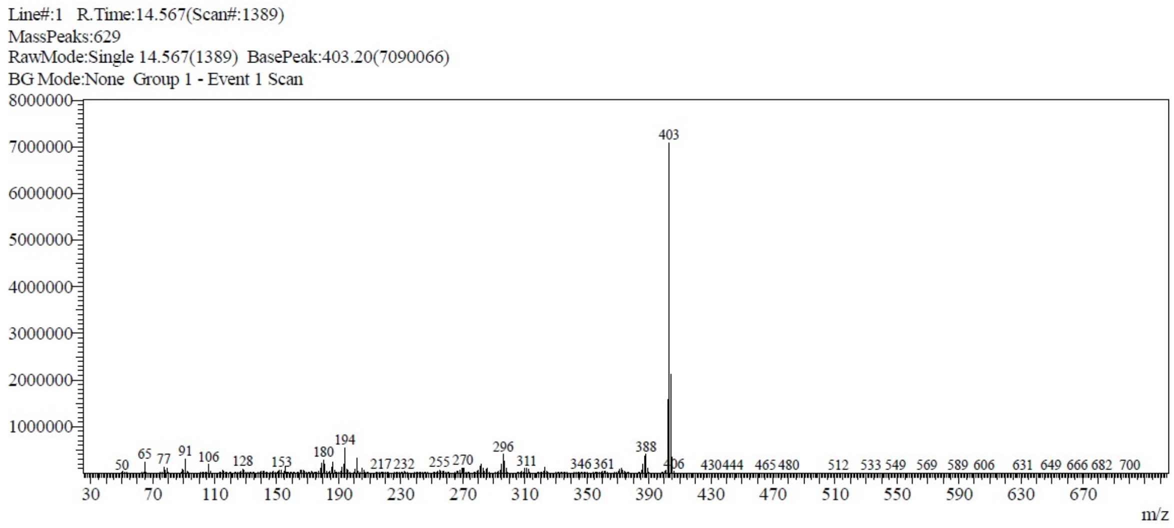

Figure **S6**. GCMS of **2**.

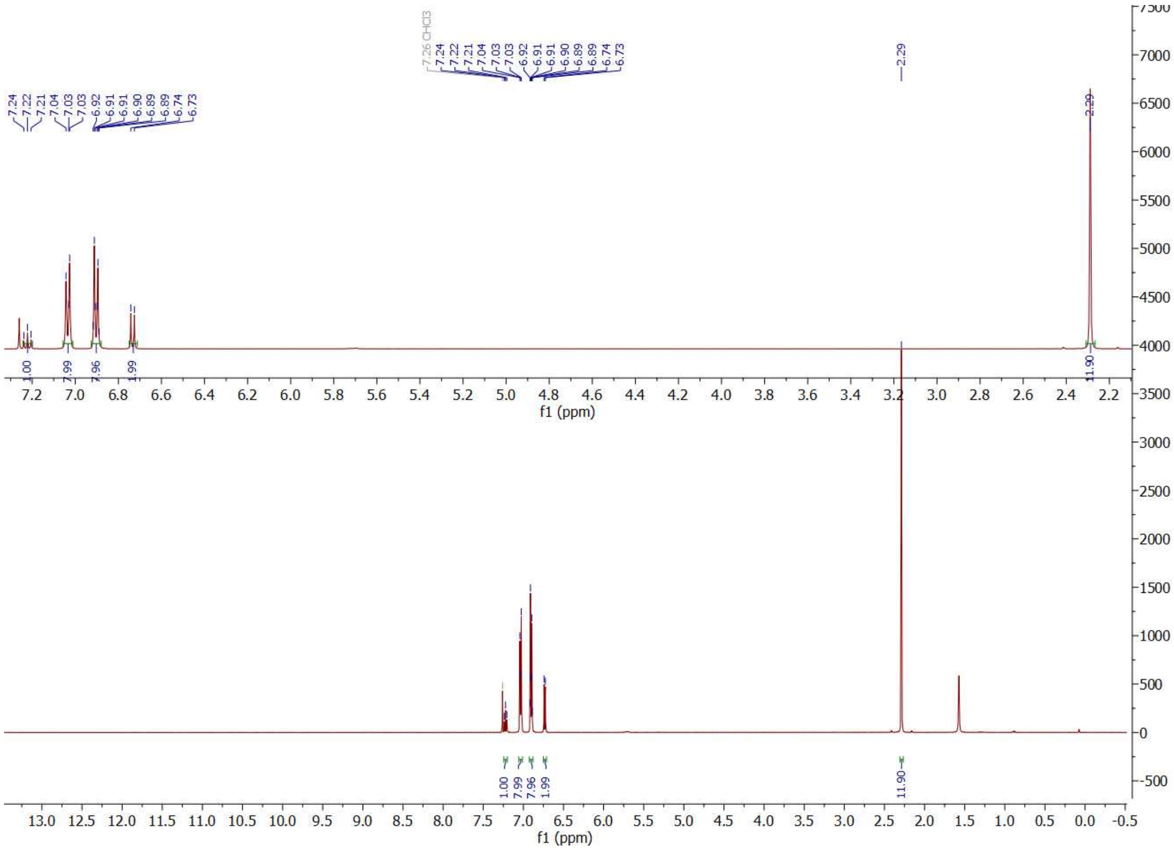

Figure **S7**. ^{1}H NMR of **4TABN1** in $CDCl_3$.

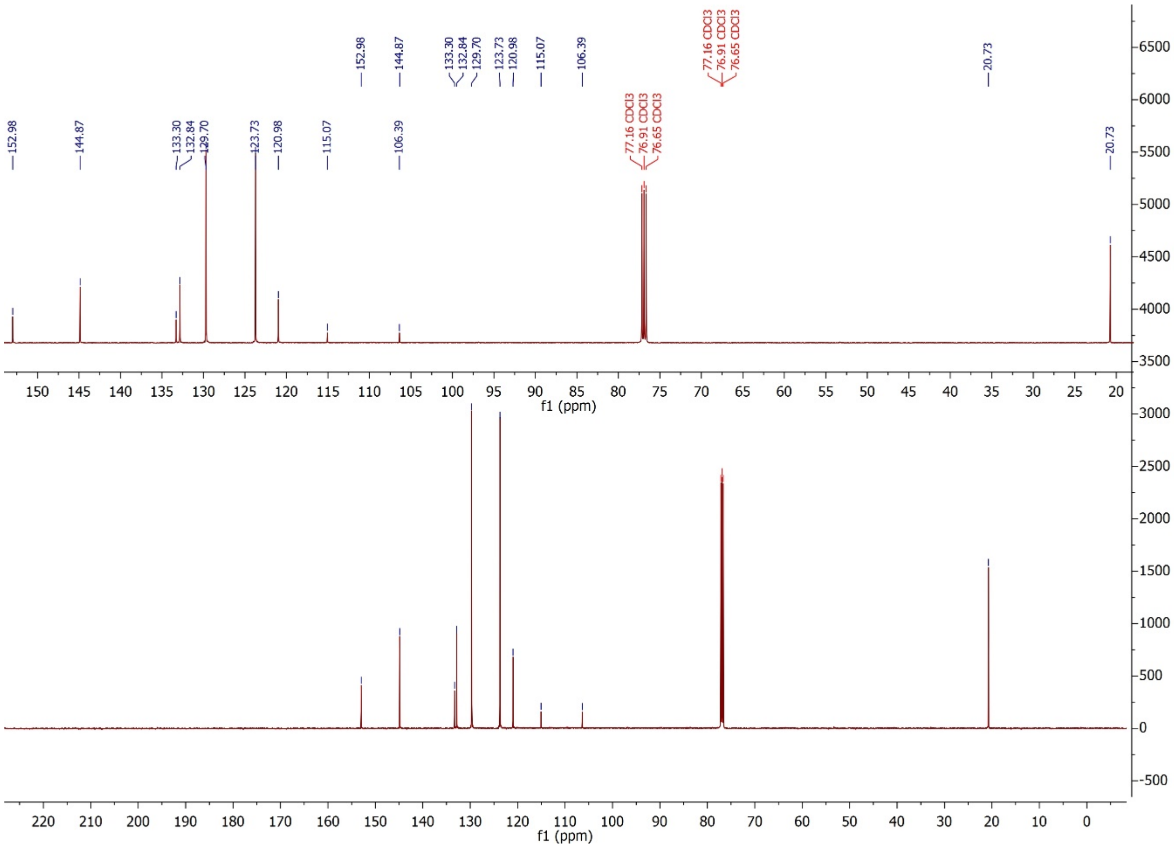

Figure **S8**. ^{13}C NMR of **4TABN1** in $CDCl_3$.

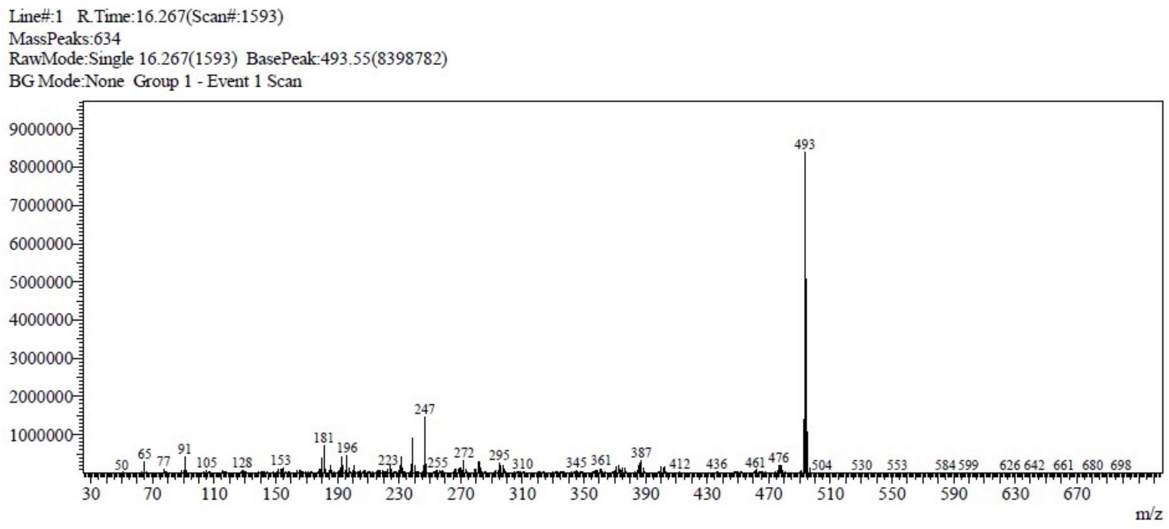

Figure **S9**. GCMS of **4TABN1**.

06/10/2020 10:15:15 Page 1 / 1

HPLC Trace Report06Oct2020

<Sample Information>

Sample Name	: SS-718		
Sample ID	:		
Method Filename	: 87% Methanol 13 Water 20 mins.lcm		
Batch Filename	: 05102020.lcb		
Vial #	: 1-47	Sample Type	: Unknown
Injection Volume	: 10 uL		
Date Acquired	: 05/10/2020 14:45:51	Acquired by	: System Administrator
Date Processed	: 05/10/2020 15:05:55	Processed by	: System Administrator

<Chromatogram>

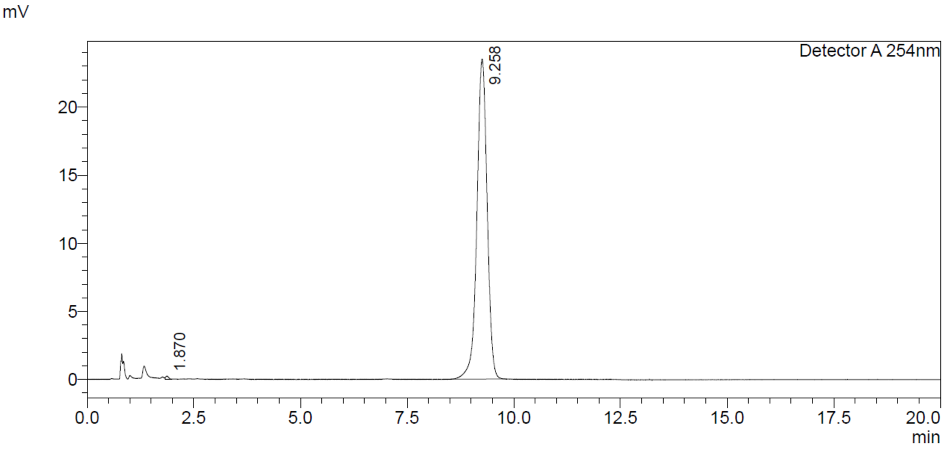

<Peak Table>

Detector A 254nm

Peak#	Ret. Time	Area	Height	Area%	Area/Height	Width at 5% Height
1	1.870	1060	221	0.269	4.784	--
2	9.258	393139	23487	99.731	16.738	0.581
Total		394199	23709	100.000		

Figure **S10**. HPLC trace of **4TABN1**.

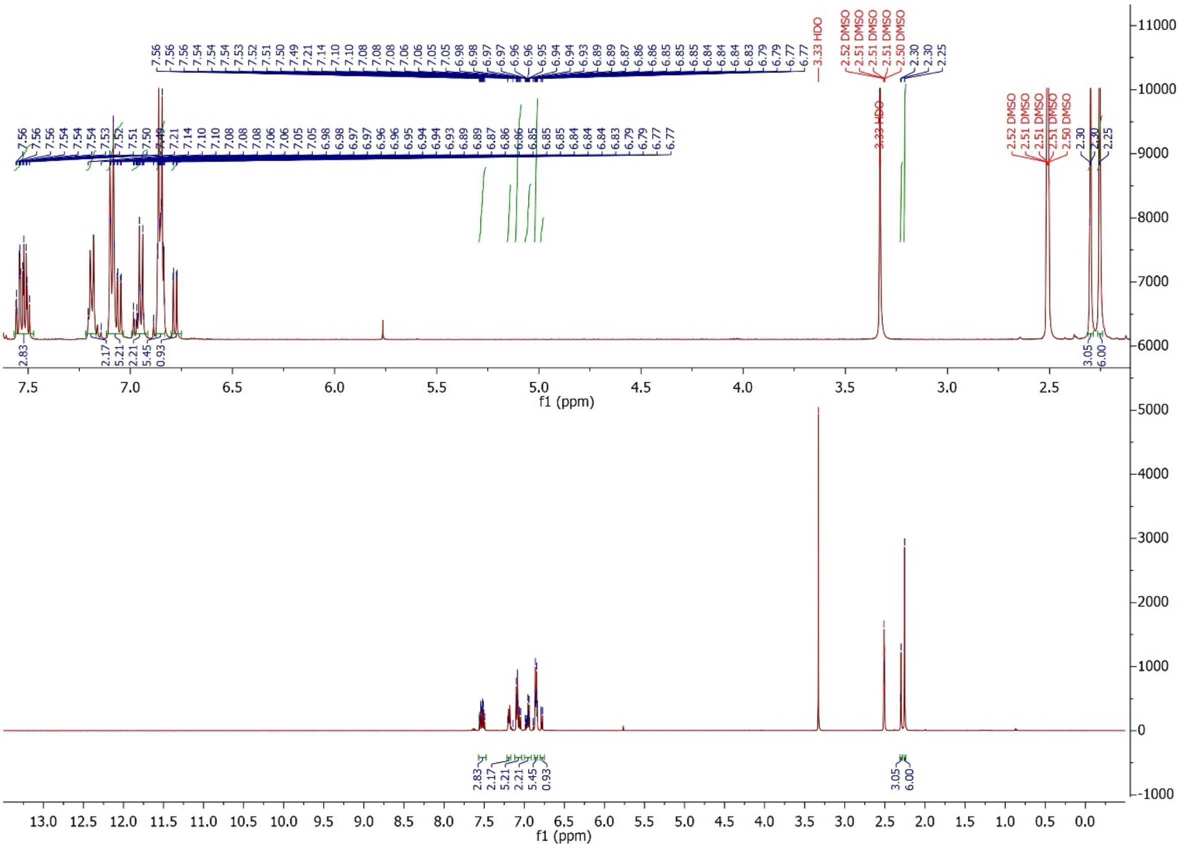

Figure **S11**. ^{1}H NMR of **3** in DMSO-d$_6$.

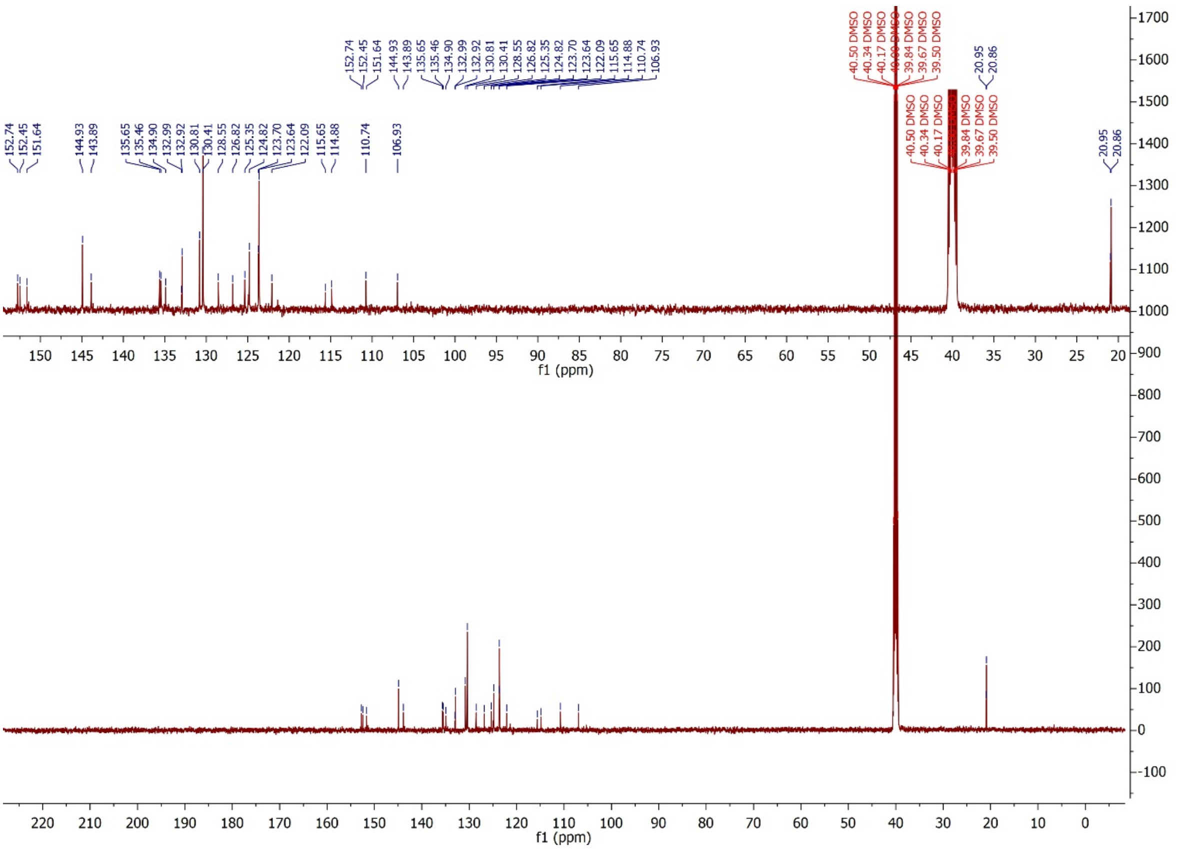

Figure **S12**. ^{13}C NMR of **3** in DMSO-d$_6$.

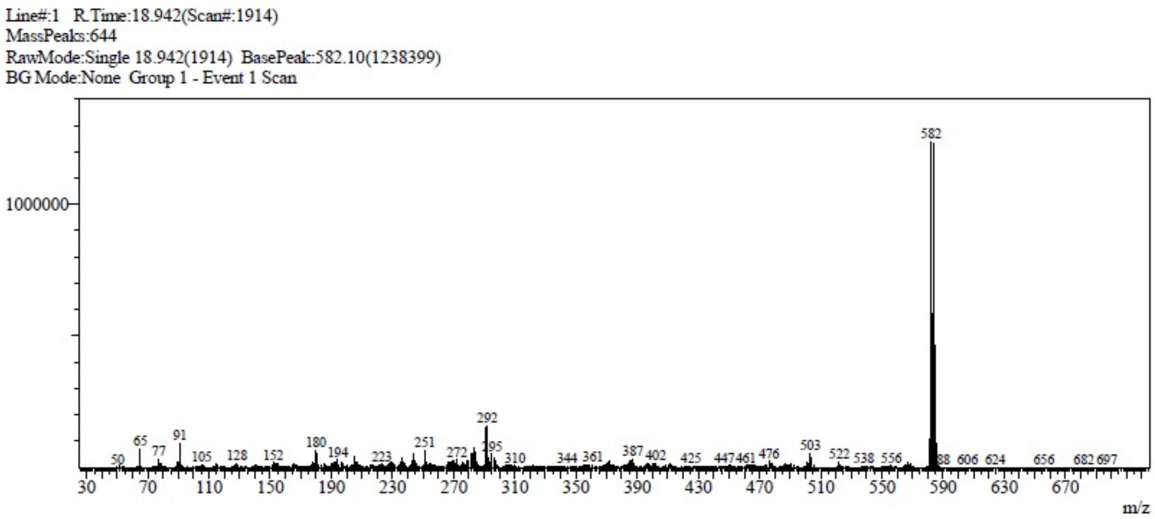

Figure **S13**. GCMS of **3**.

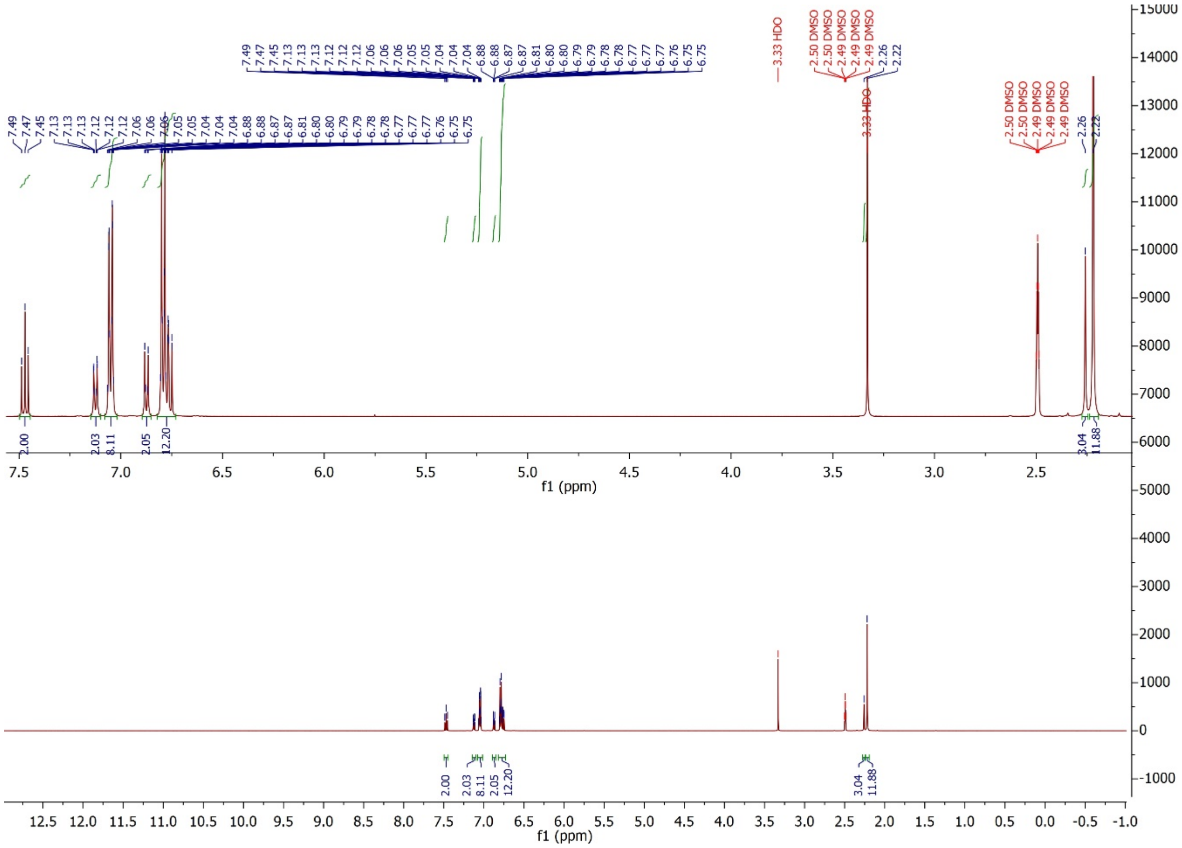

Figure **S14**. ^{1}H NMR of **5TABN2** in DMSO-d_6.

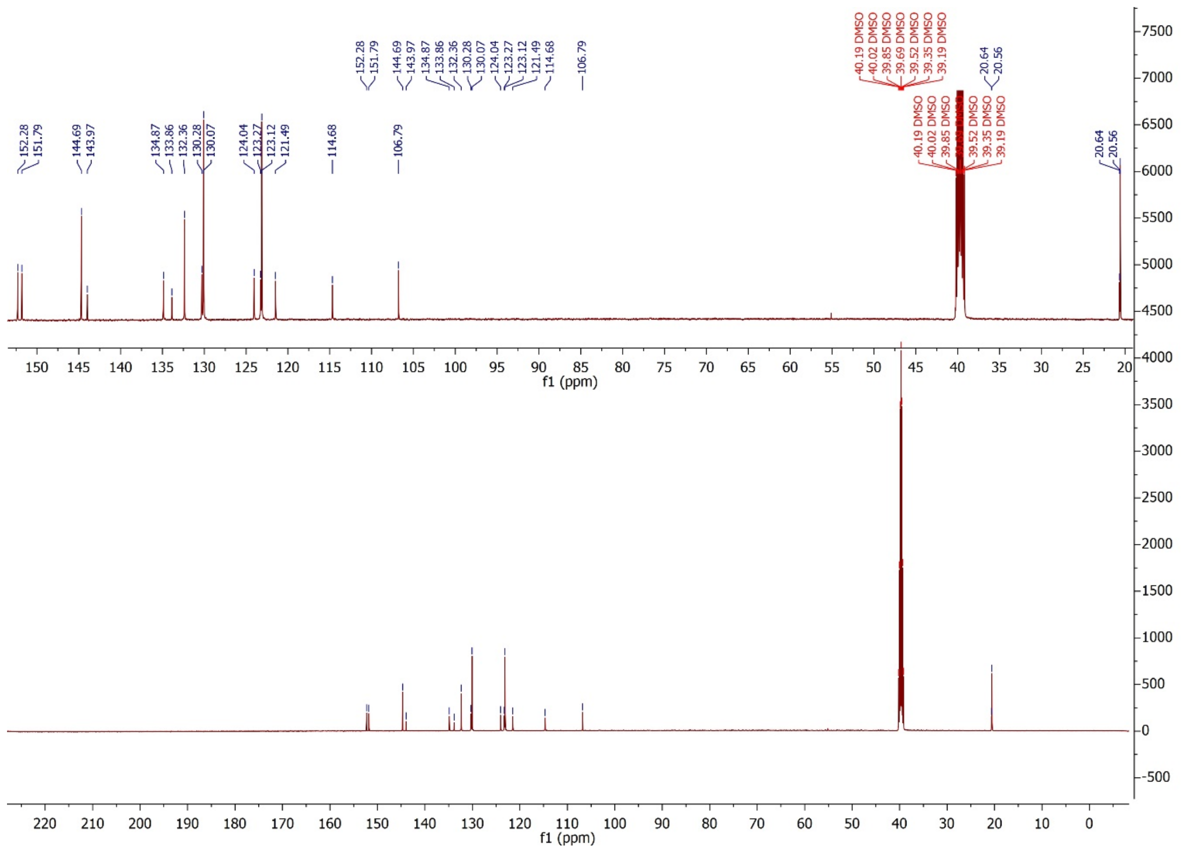

Figure **S15**. ^{13}C NMR of **5TABN2** in DMSO-d_6.

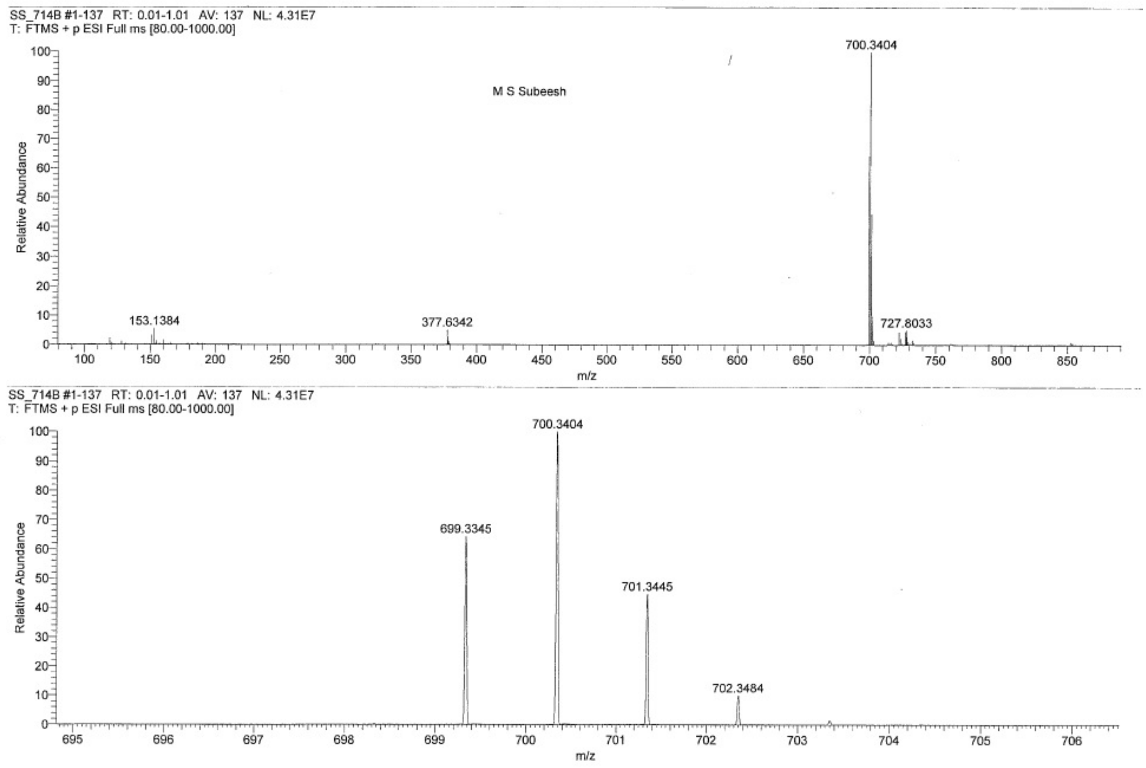

Figure **S16**. HRMS of **5TABN2**.

HPLC Trace Report05Oct2020

<Sample Information>

Sample Name	: SS-902		
Sample ID	:		
Method Filename	: 88% Methanol 12 Water 20 mins.lcm		
Batch Filename	: 05102020.lcb		
Vial #	: 1-48	Sample Type	: Unknown
Injection Volume	: 10 uL		
Date Acquired	: 05/10/2020 15:51:25	Acquired by	: System Administrator
Date Processed	: 05/10/2020 16:11:27	Processed by	: System Administrator

<Chromatogram>

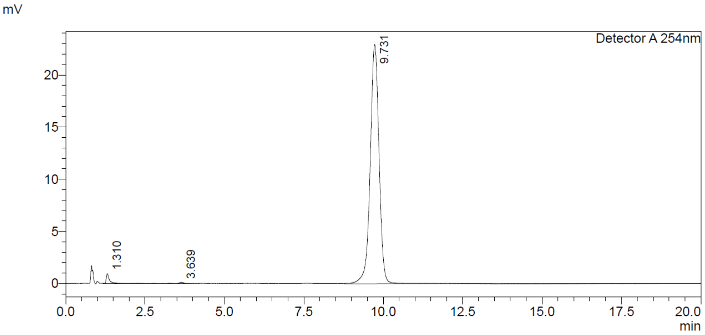

<Peak Table>

Detector A 254nm

Peak#	Ret. Time	Area	Height	Area%	Area/Height	Width at 5% Height
1	1.310	11195	981	2.461	11.409	0.468
2	3.639	1887	149	0.415	12.651	--
3	9.731	441792	22925	97.124	19.271	0.670
Total		454875	24055	100.000		

Figure **S17**. HPLC trace of **5TABN2**.

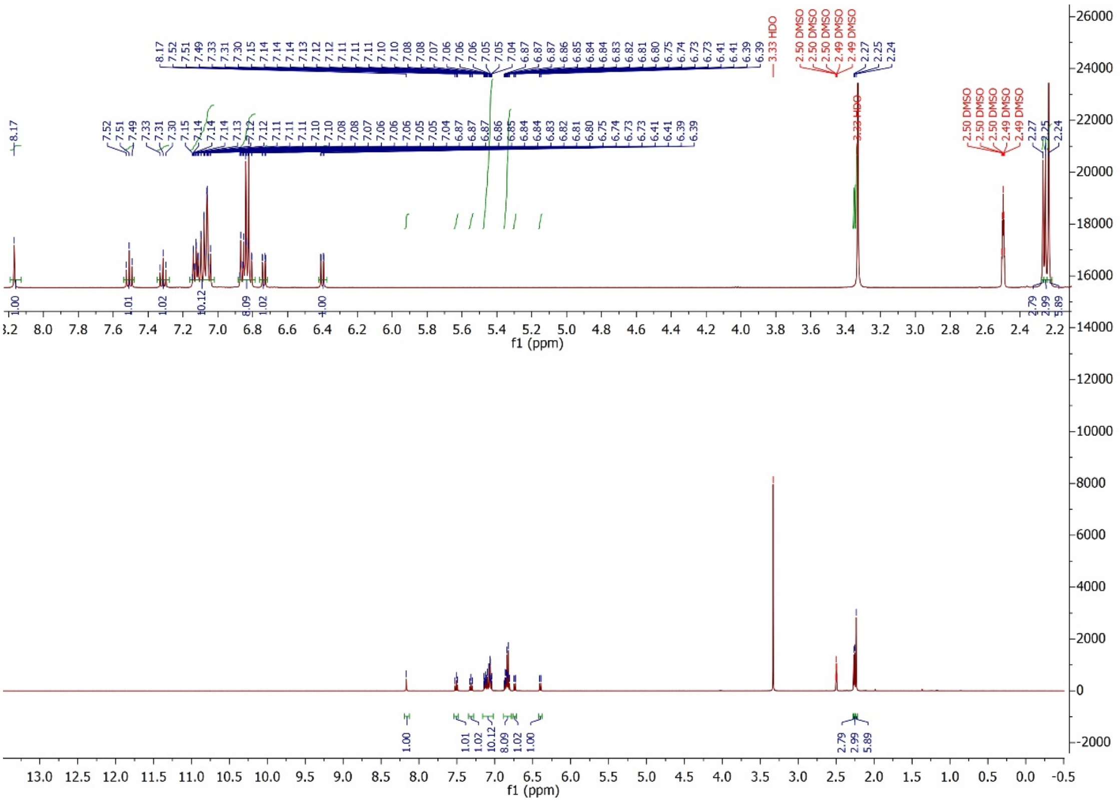

Figure **S18**. ^{1}H NMR of **4** in DMSO-d6.

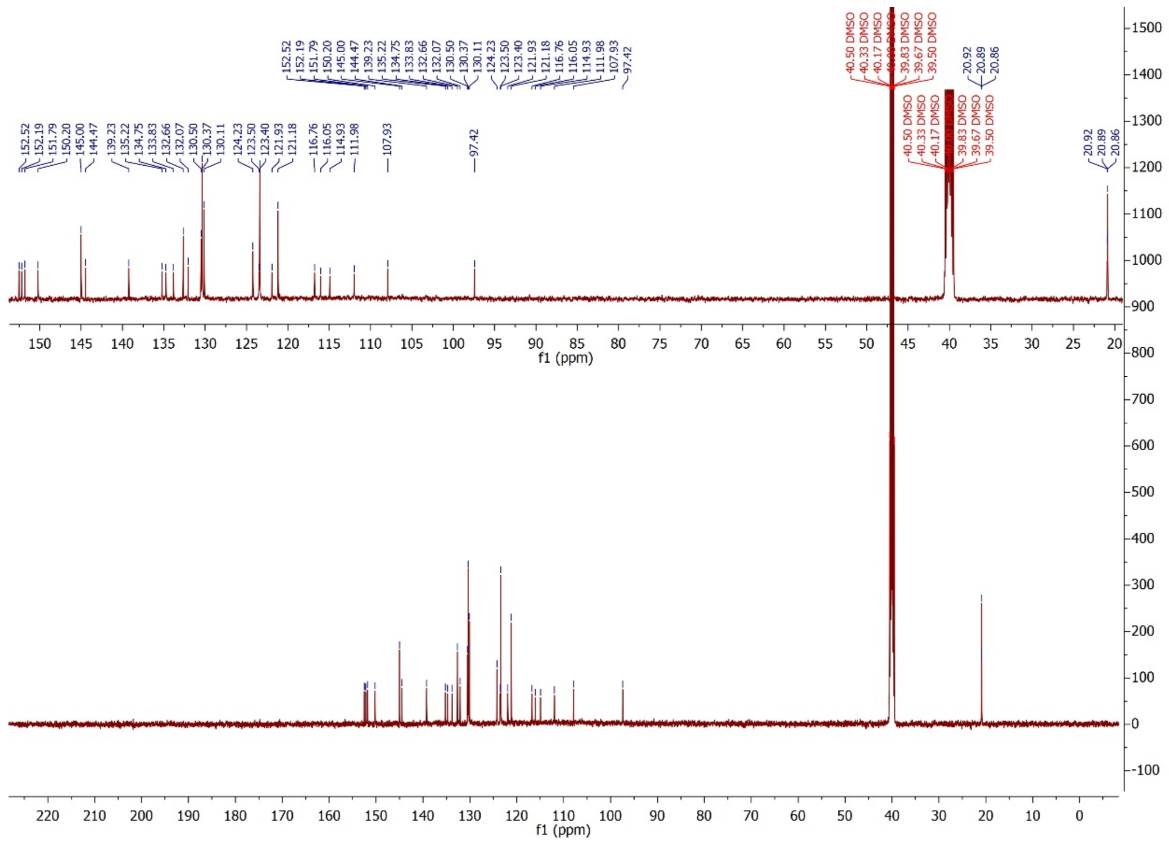

Figure **S19**. ^{13}C NMR of **4** in DMSO-d6.

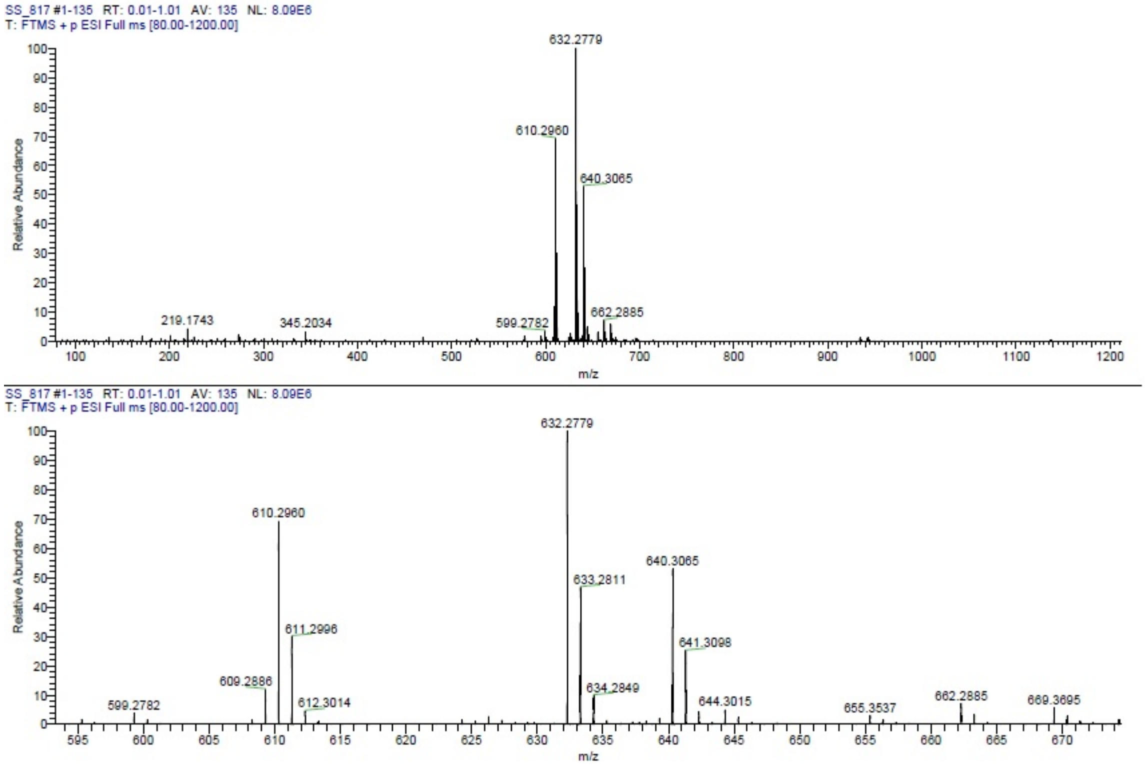

Figure **S20**. HRMS of **4**.

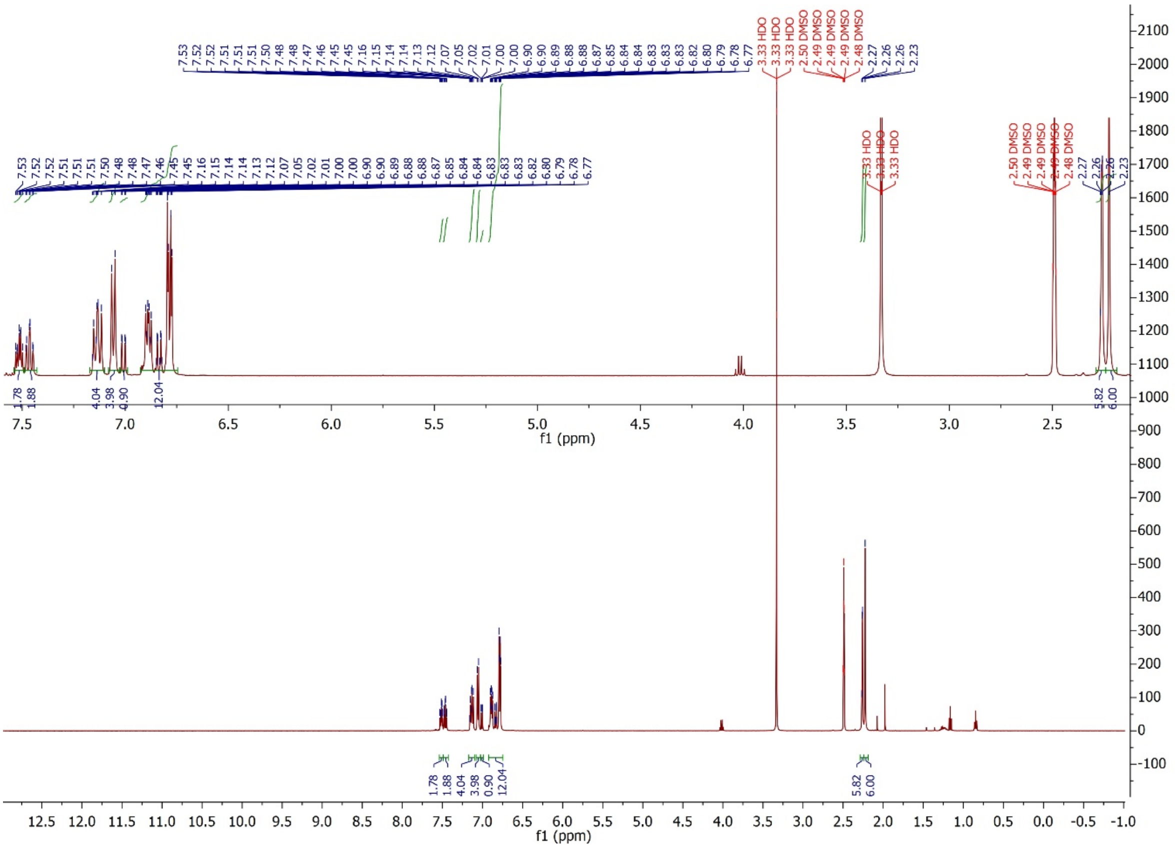

Figure **S21**. ^{1}H NMR of **5** in DMSO-d_6.

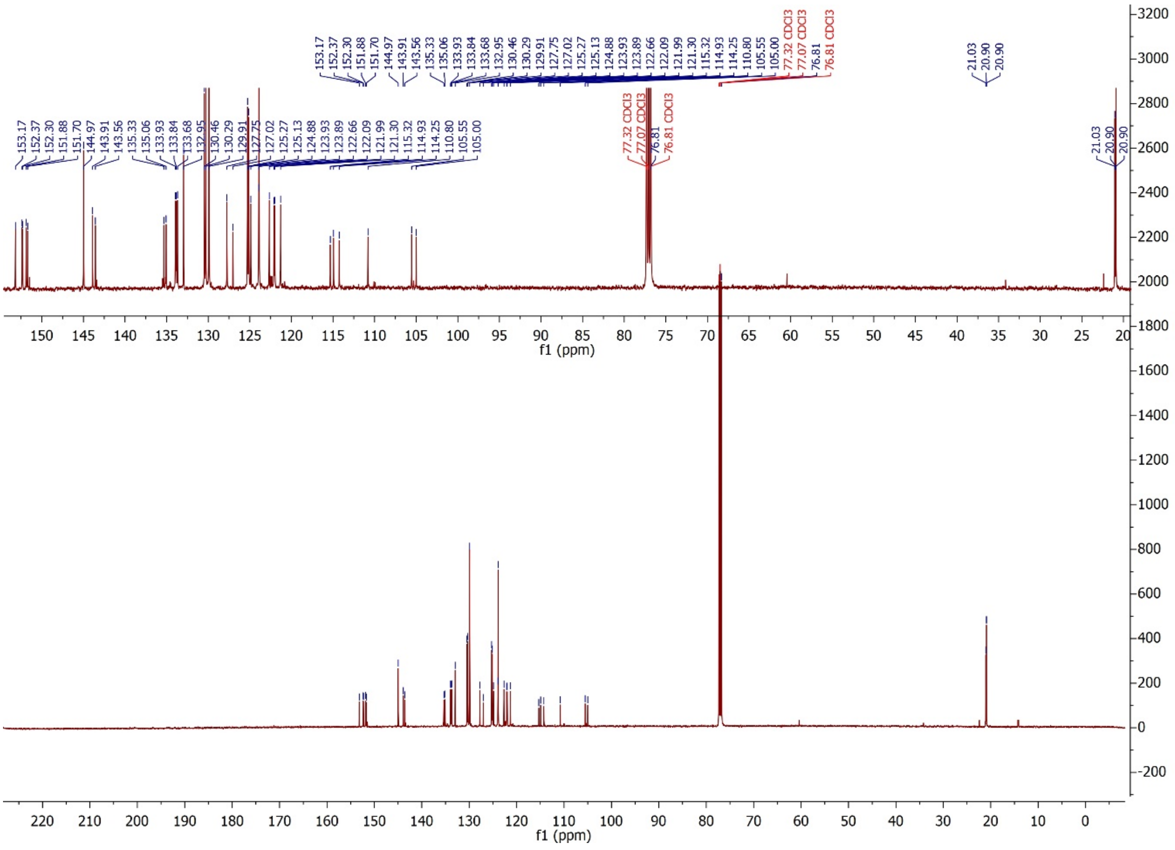

Figure **S22**. ^{13}C NMR of **5** in $CDCl_3$.

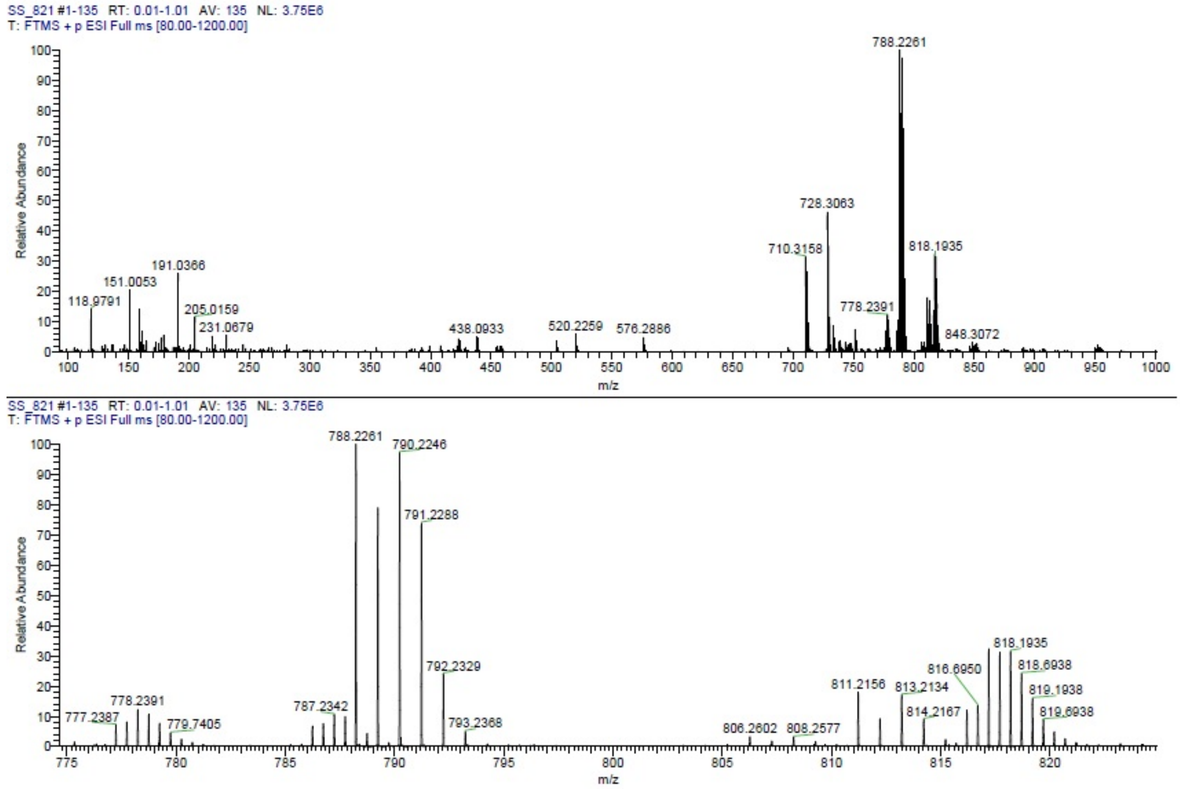

Figure **S23**. HRMS of **5**.

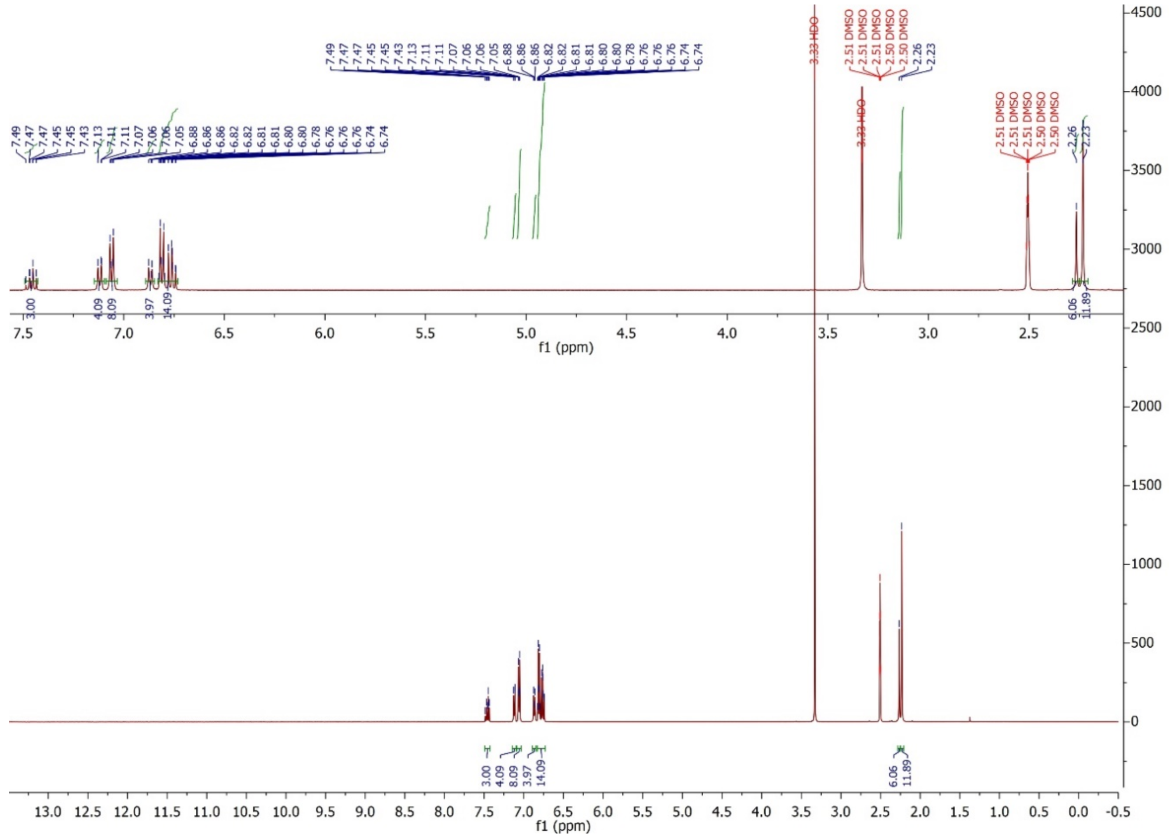

Figure **S24**. ^{1}H NMR of **6TABN3** in DMSO-d_6.

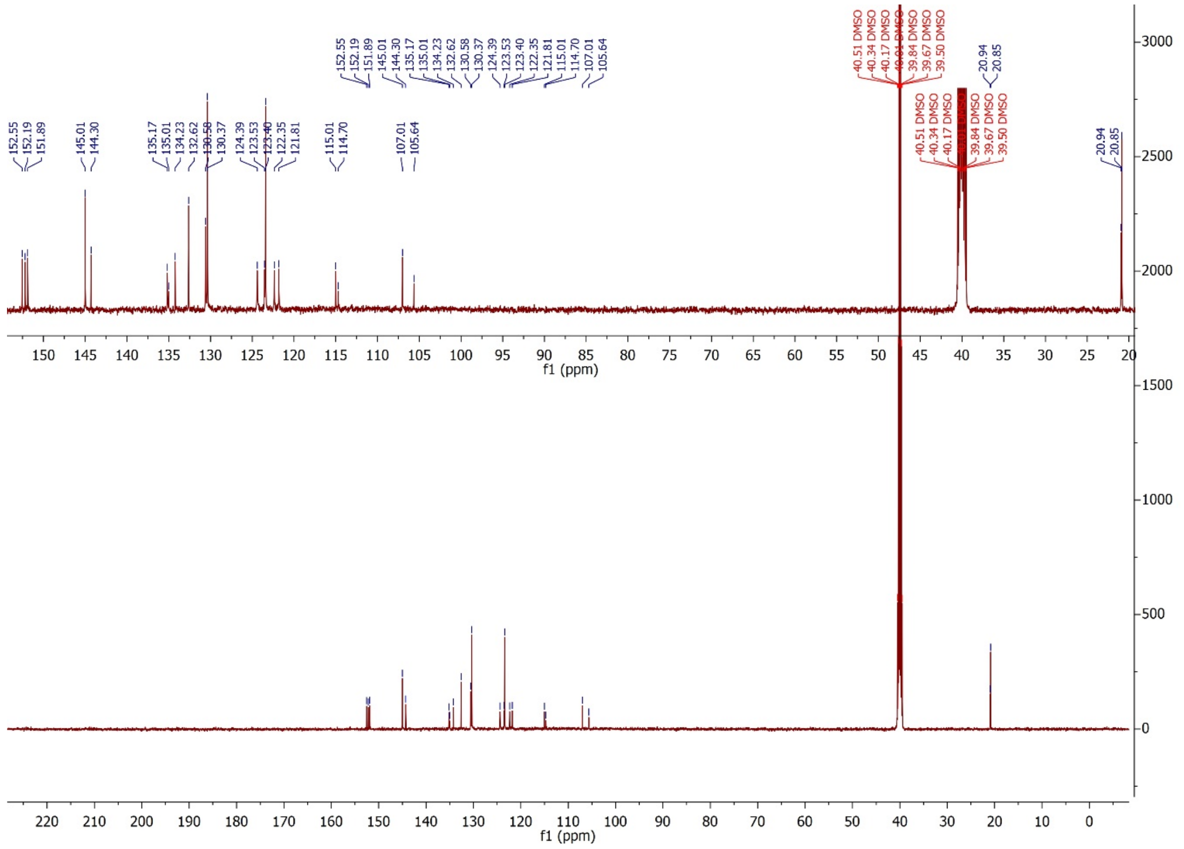

Figure **S25**. ^{13}C NMR of **6TABN3** in DMSO-d$_6$.

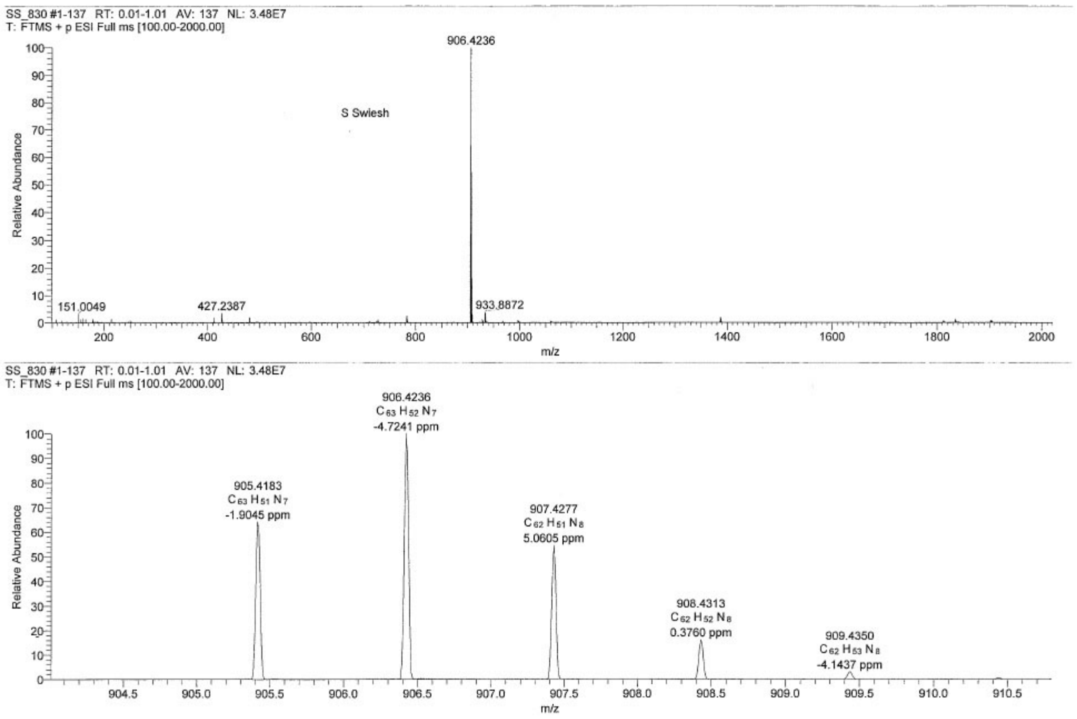

Figure **S26**. HRMS of **6TABN3**.

HPLC Trace Report14Aug2020

<Sample Information>

Sample Name	: SS-830		
Sample ID	:		
Method Filename	: 90% Methanol 10 Water 20 mins.lcm		
Batch Filename	: 16072020.lcb		
Vial #	: 1-50	Sample Type	: Unknown
Injection Volume	: 10 uL		
Date Acquired	: 14/08/2020 16:14:00	Acquired by	: System Administrator
Date Processed	: 14/08/2020 16:34:03	Processed by	: System Administrator

<Chromatogram>

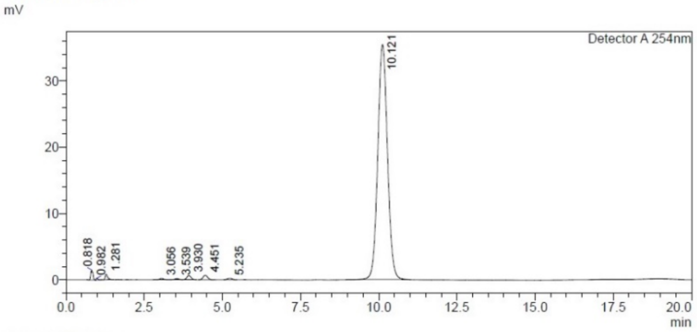

<Peak Table>

Detector A 254nm

Peak#	Ret. Time	Area	Height	Area%	Area/Height	Width at 5% Height
1	0.818	7073	1437	0.892	4.923	0.137
2	0.982	1011	248	0.127	4.071	0.155
3	1.281	4476	823	0.564	5.440	0.194
4	3.056	1449	189	0.183	7.666	0.224
5	3.539	1331	154	0.168	8.645	0.261
6	3.930	5842	610	0.737	9.571	0.306
7	4.451	7137	681	0.900	10.481	0.343
8	5.235	2303	212	0.290	10.871	0.309
9	10.121	762354	35437	96.138	21.513	0.720
Total		792976	39791	100.000		

Figure **S27**. HPLC trace of **6TABN3**.

Electrochemistry

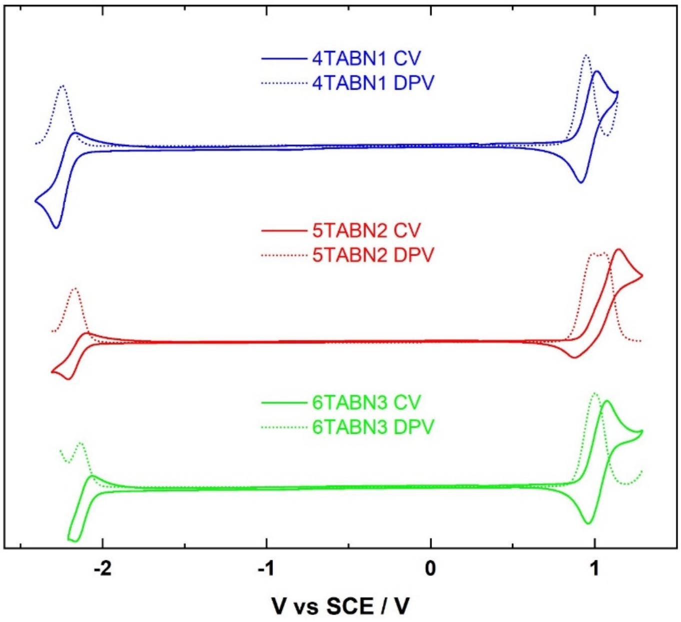

Figure **S28.** Cyclic (CV) and differential pulse voltammograms (DPV) measured in degassed MeCN with 0.1 M [*n*Bu_4N]PF_6 as the supporting electrolyte and Fc/Fc^+ as the internal reference (0.38 V vs SCE).[1] Scan rate = 100 mV s^{-1}.

Cyclic voltammetry (CV) and differential pulse voltammetry (DPV) measurements were carried out (Figure **S28**) to determine the energies of the HOMO and LUMO levels. The amine-based oxidation potentials (E^{ox}), extracted from the peak value of first oxidation wave of the DPVs, are 0.95 V, 0.99 V, and 1.0 V vs SCE, respectively, for **4TABN1**, **5TABN2** and **6TABN3**. The corresponding HOMO energy levels were calculated to be -5.75 eV, -5.79 eV, and -5.80 eV, respectively, for **4TABN1**, **5TABN2** and **6TABN3**. **4TABN1** and **6TABN3** show reversible oxidations waves while for **5TABN2** it is quasi-reversible; **5TABN2** also shows a second distinct oxidation process at 1.10 V, reflecting the two different amine donors, while the two electronically distinct amine donors in **6TABN3** are not discernible by DPV. The benzonitrile-localized reduction potentials (E^{red}), determined from the peak value of first reduction wave of the DPVs, are -2.25 V, -2.17 V, and -2.14 V, respectively, corresponding to LUMO levels of -2.55 eV, -2.63 eV, and -2.66 eV for **4TABN1**, **5TABN2** and **6TABN3**. The cathodic shift of E^{red} for **4TABN1** reflects the relatively increased conjugation of the donor groups with the BN acceptor in this compound as compared to **5TABN2** and **6TABN3**. The corresponding energy gaps were found to be 3.2 eV, 3.16 eV and 3.14 eV, respectively, for **4TABN1**, **5TABN2** and **6TABN3**, revealing only a modest decrease in the HOMO-LUMO gap with increasing number of repeat units. The trends in both decreasing HOMO and LUMO with the corresponding decreasing ΔEs align well with the DFT calculated trends.

Photophysical Characterization

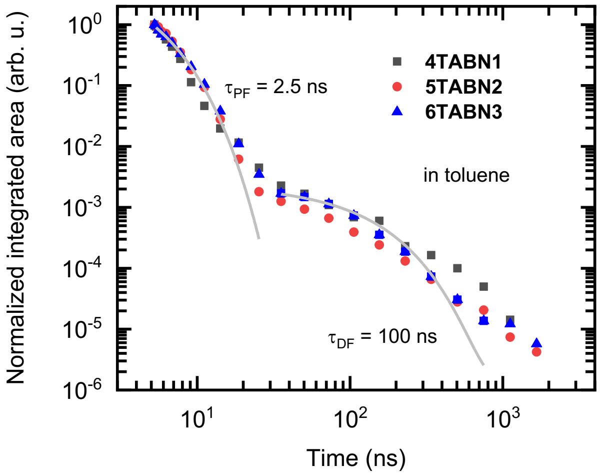

Figure **S29**. The transient PL decay of the compounds in oxygen-free toluene at RT. Integrated across the full spectral range. λ_{exc} = 355 nm.

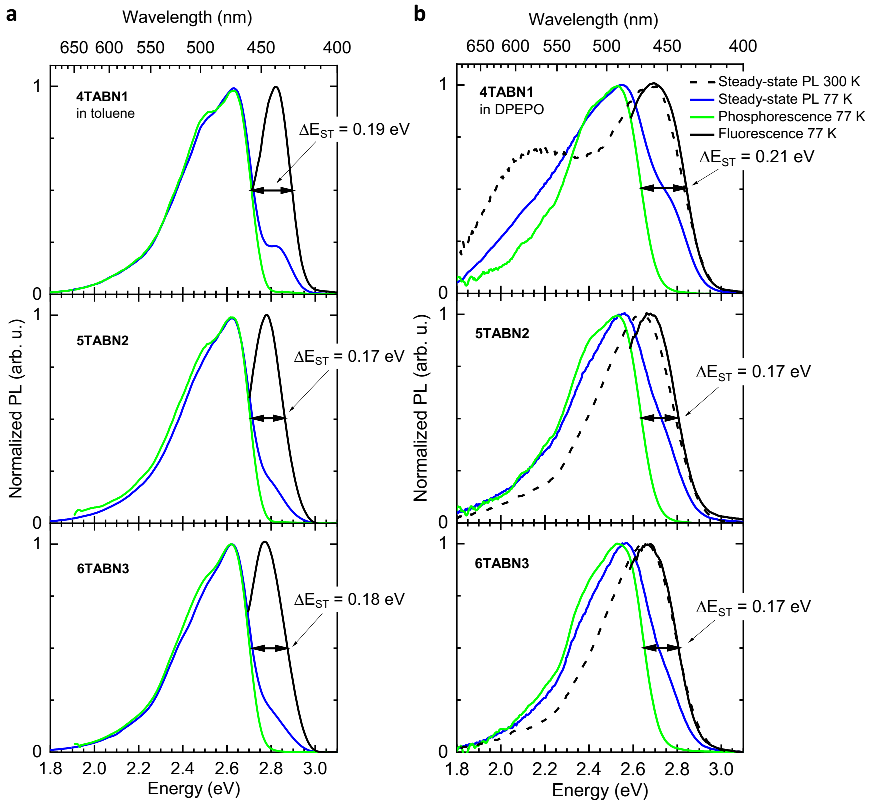

Figure **S30**. Figure 2 as in the main text but with the bottom scale in energy units. Steady-state emission (blue line), phosphorescence (delay 150 ms, gate 50 ms, green line) and fluorescence (black line) obtained as the difference between the steady-state emission and phosphorescence at 77 K in (a) toluene solution and (b) 10 wt% film in DPEPO. The black dashed line represents the steady state emission at 300 K. λ_{exc} = 300 nm.

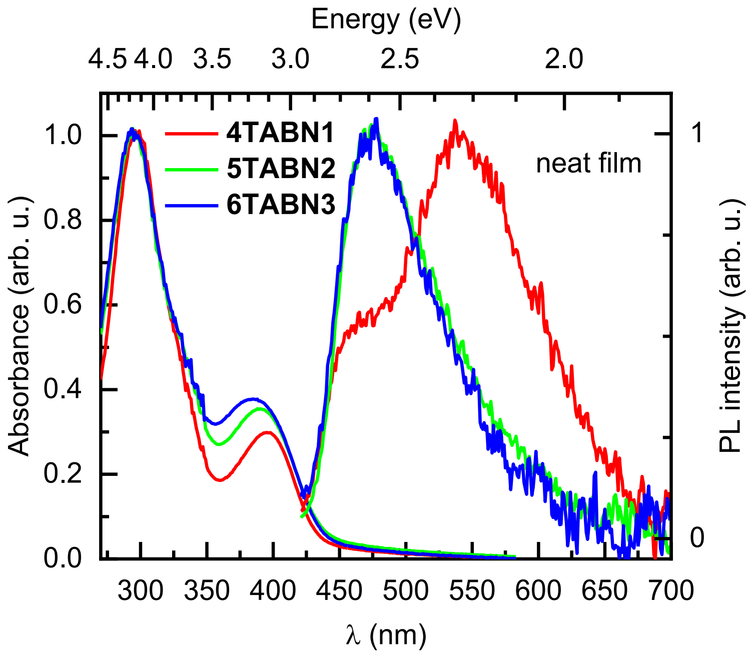

Figure **S31**. The absorption and steady-state PL emission of neat films at RT. λ_{exc} = 315 nm.

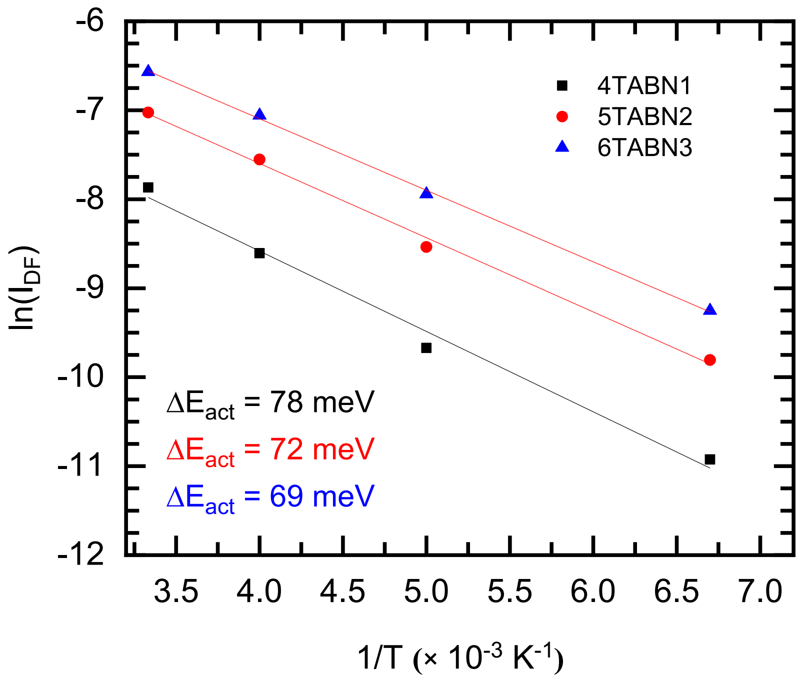

Figure **S32**. Activation energies of **4TABN1**, **5TABN2** and **6TABN3** 3 wt% films in DPEPO.

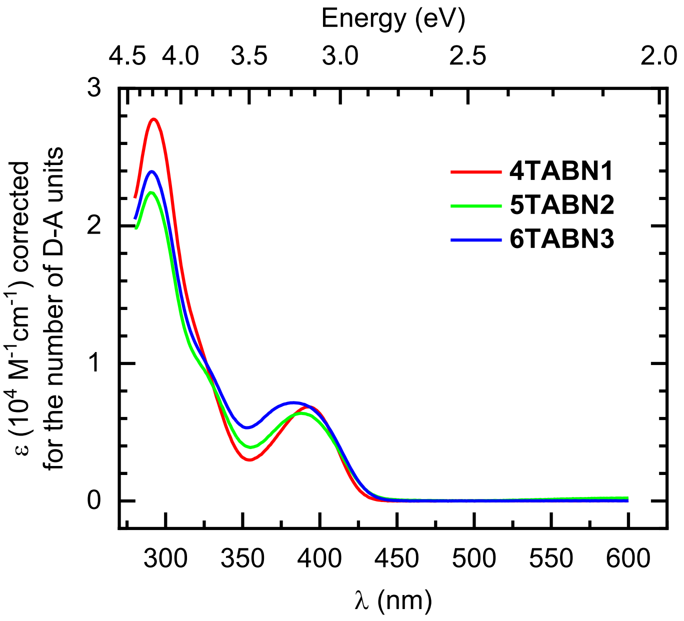

Figure **S33**. Extinction coefficients as presented in Figure **3** in the main text divided by 1.5, 2.5, and 3.5 for **4TABN1**, **5TABN2** and **6TABN3**, respectively.

Table **S1**. Summary of photophysical parameters for **4TABN1**, **5TABN2** and **6TABN3** in DPEPO (10 wt%) film. The delayed lifetime is fitted using a mono-exponential to estimate the fastest RISC component.

	Φ_{PL}[(a)] (%)	Φ_{DF}/Φ_{PF}[(b)]	τ_{PF}[(c)] (ns)	τ_{DF}[(d)] (µs)	k_r^S[(e)] ($\times10^7$ s^{-1})	Φ_{ISC}[(f)]	k_{ISC}[(g)] ($\times10^8$, s^{-1})	k_{IC}^S[(h)] ($\times10^7$ s^{-1})	k_{RISC}[(i)] ($\times10^5$ s^{-1})
**4TABN1**	35	0.53	11.0	8.4	2.1	0.36	0.3	3.7	1.8
**5TABN2**	35	1.99	7.0	7.9	1.7	0.75	1.1	1.9	3.4
**6TABN3**	30	2.49	7.0	7.7	1.2	0.77	1.1	2.1	4.2

[a] total Φ_{PL} (λ_{exc} = 315 nm); [b] ratio of the integrated areas of delayed and prompt emission; [c] lifetime of prompt emission estimated using a mono-exponential fit at 300 K; [d] lifetime of delayed emission as estimated using a mono-exponential fit 300 K; [e] radiative decay rate constant of the singlet excitons; [f] intersystem crossing yield; [g] intersystem crossing rate constant; [h] internal conversion rate constant of the singlet excitons; [i] reverse-intersystem crossing rate constant (fast component, corresponds to around 60% of the total delayed emission).

Quantum chemical calculations

Table **S2**. Ground state properties of each emitter at PBE0/6-31G(d,p).

Compound	HOMO / eV	LUMO / eV	Δ*E* / eV
**4TABN1**	-5.17	-1.07	4.10
**5TABN2**	-5.27	-1.39	3.88
**6TABN3**	-5.32	-1.54	3.78

Table **S3**. Excited state properties of each emitter at their lowest energy ground state conformation at TDA-PBE0/6-31G(d,p).

Compound	S_1 (φ_S) / eV	*f*	T_1 (φ_S) / eV	T_2 (φ_S) / eV	T_3 (φ_S) / eV	T_4 (φ_S) / eV	ΔE_{ST} / eV
**4TABN1**	3.27 (0.55)	0.25	2.90 (0.58)	2.94 (0.75)	3.29 (0.78)	3.46 (0.85)	0.37
**5TABN2**	3.11 (0.52)	0.41	2.81 (0.58)	2.87 (0.69)	2.88 (0.65)	3.25 (0.81)	0.30
**6TABN3**	3.05 (0.51)	0.56	2.79 (0.56)	2.82 (0.64)	2.85 (0.64)	2.86 (0.66)	0.26

Where φ_S is an indication of overlap calculated using the NANCYX 2.0 package, with a value of 1 = locally excited (LE) nature and a value of 0 = charge transfer (CT).

Table **S4**. Singlet excited state energies and oscillator strengths using various starting geometries using TDA-PBE0/6-31G(d,p).

Compound	S_0 optimized geometry		S_1 optimized geometry		S_0 optimized geometry with toluene	
	**Energy / eV**	**f**	**Energy / eV**	**f**	**Energy / eV**	**f**
**4TABN1**	3.27	0.25	2.26	0.0025	3.19	0.3119
**5TABN2**	3.11	0.41	2.15	0.0028	3.04	0.4587
**6TABN3**	3.05	0.56	2.21	0.0018	2.99	0.6229

Table **S5**. Spin orbit coupling between S_1 and triplet excited states calculated at their respective lowest energy ground state conformation at TDA-PBE0/6-31G(d,p).

**Compound**	**T_1 / cm^{-1}**	**T_2 / cm^{-1}**	**T_3 / cm^{-1}**	**T_4 / cm^{-1}**
**4TABN1**	0.013	1.208	0.407	0.742
**5TABN2**	0.140	0.674	0.600	1.414
**6TABN3**	0.027	0.478	0.489	0.480

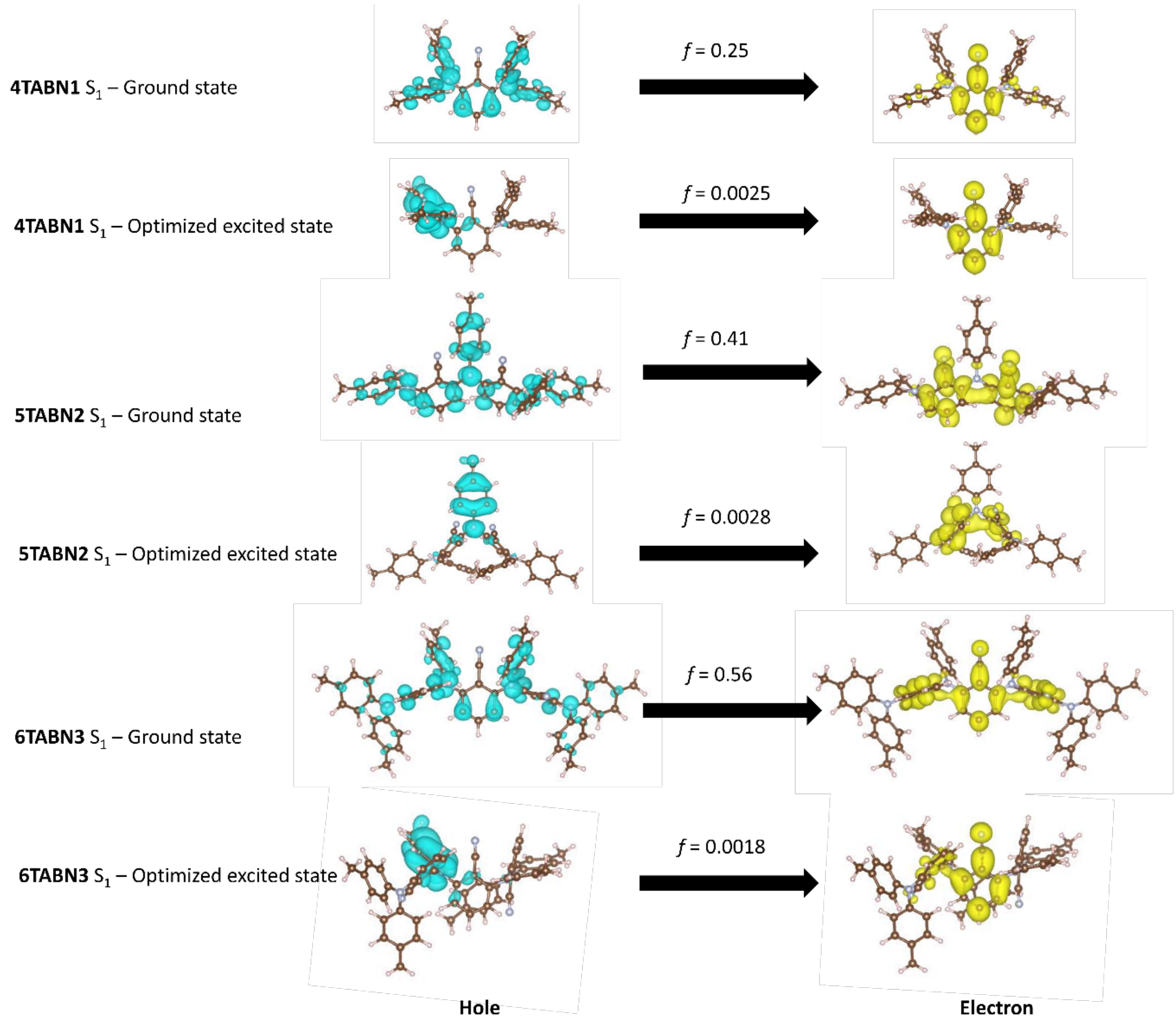

Figure **S34**. Hole and electron plots of the S_1 excited state of **4TABN1**, **5TABN2** and **6TABN3** at the ground and optimized S_1 states using TDA-PBE0/6-31G(d,p).

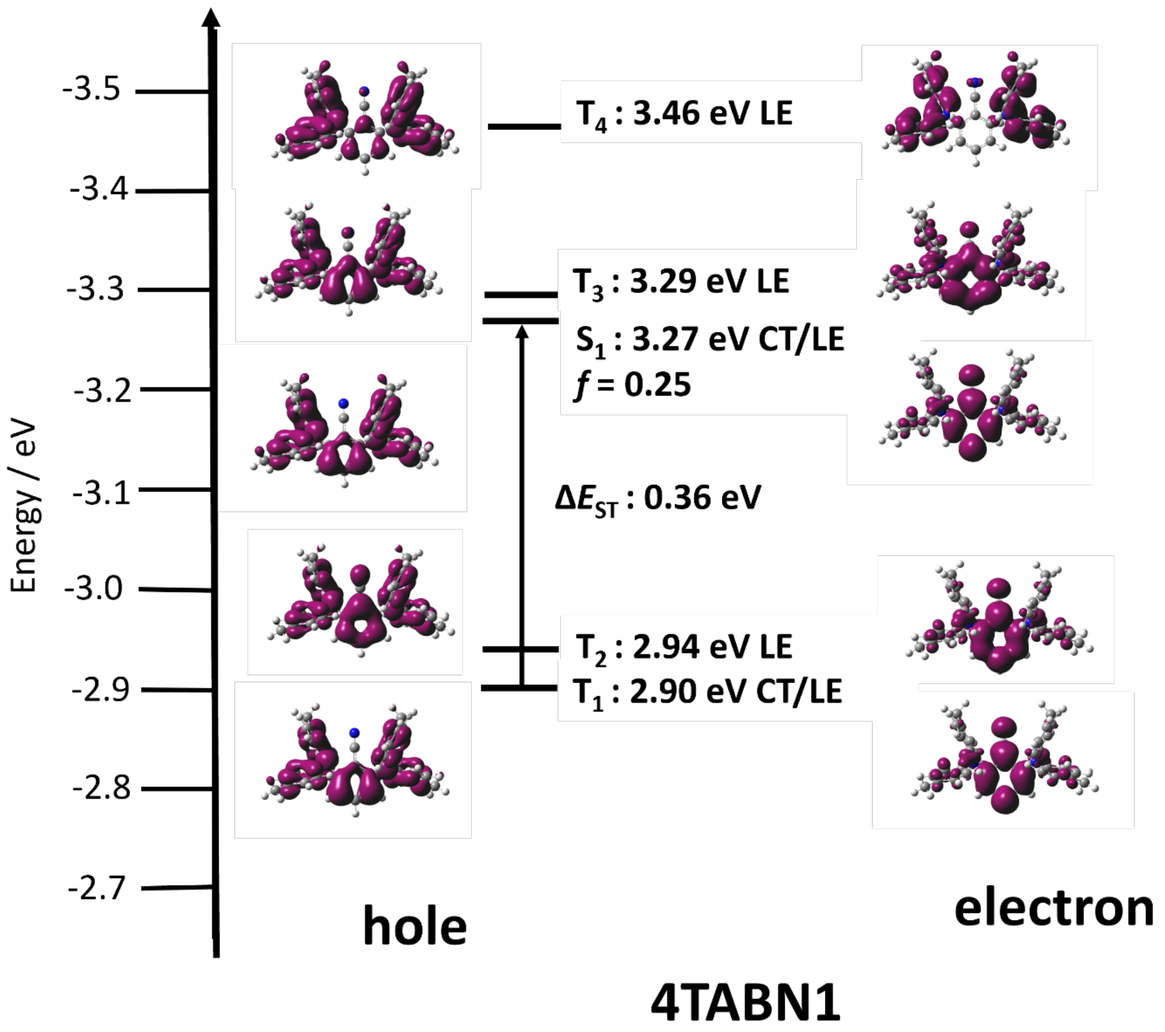

Figure **S35**. Hole/electron plots of **4TABN1** calculated at TDA-PBE0/6-31G(d,p) using the attachment detachment formalism, where *f* = oscillator strength.

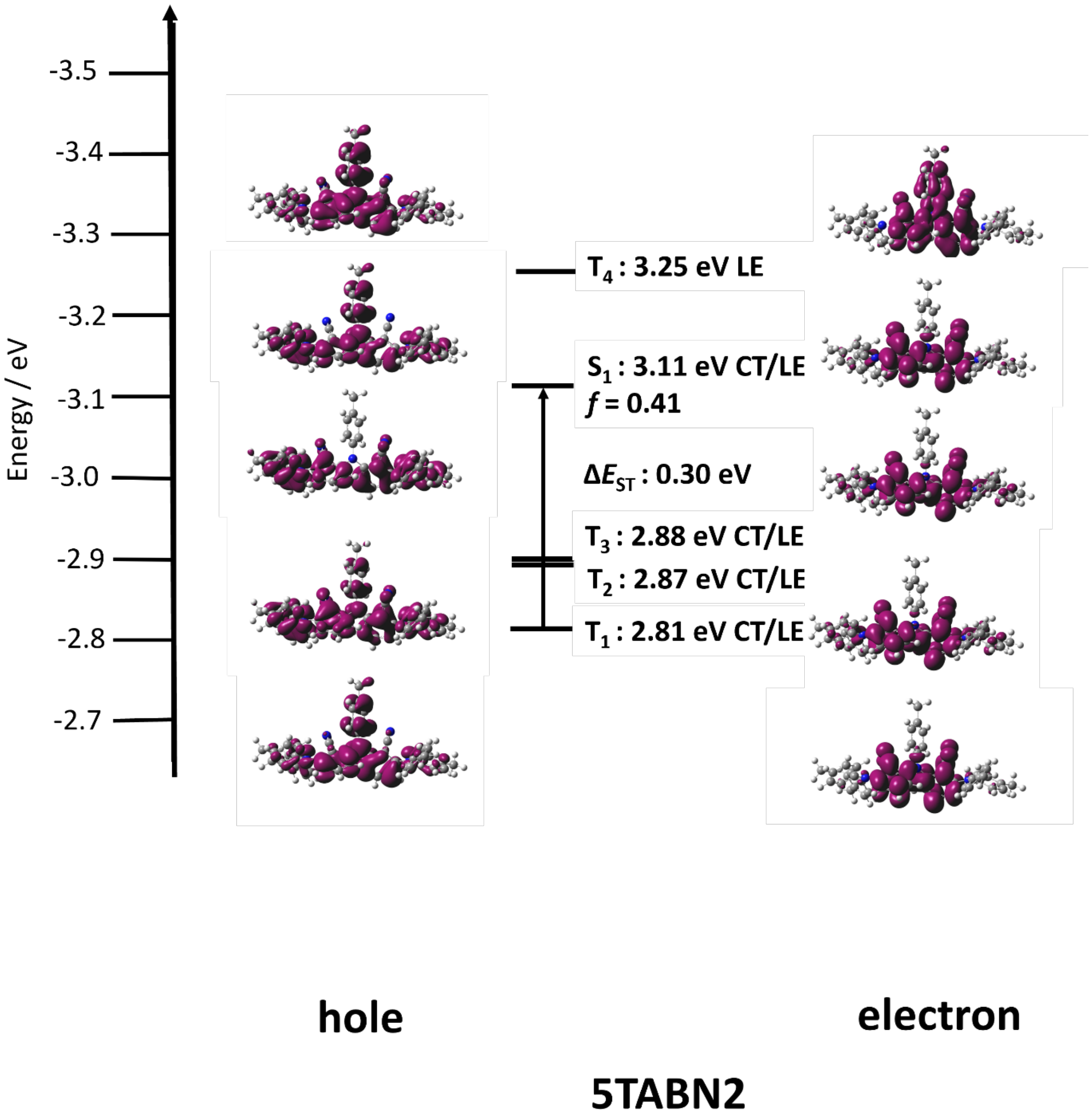

Figure **S36**. Hole/electron plots of **5TABN2** calculated at TDA-PBE0/6-31G(d,p) using the attachment detachment formalism, where *f* = oscillator strength.

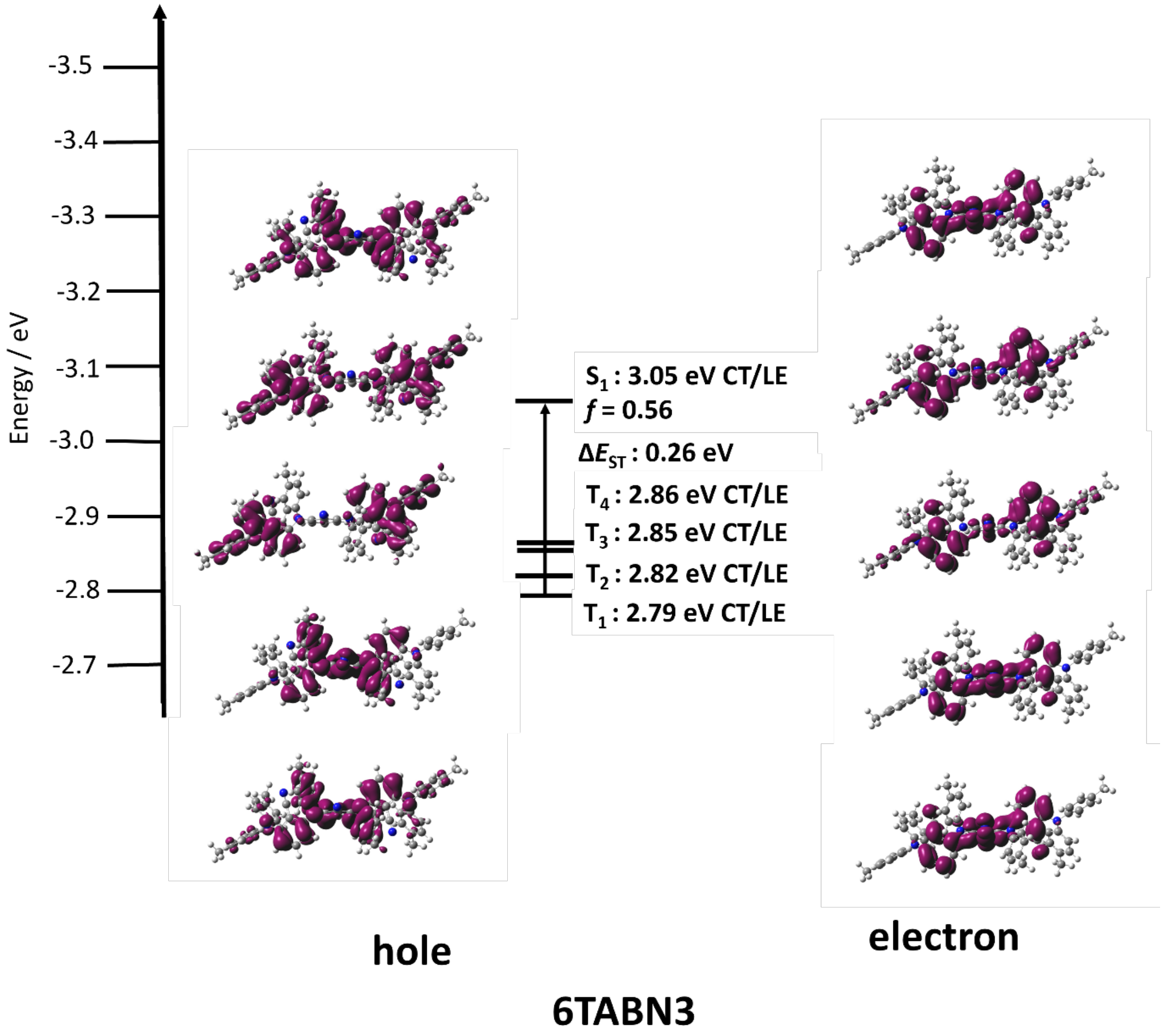

Figure **S37**. Hole/electron plots of **6TABN3** calculated at TDA-PBE0/6-31G(d,p) using the attachment detachment formalism, where *f* = oscillator strength.

For **4TABN1** the electron density is predominately located on the BN unit in each of the low-lying excited states, save for T_4 where it is localized on the ditolylamine (DTA). The hole density is located on both the DTA units and the phenyl of BN, it is the large degree of overlap here that results in the presence of mostly mixed CT/LE or LE excited states. For both **5TABN2** and **6TABN3**, similar trends are observed with the electron density generally localized on the BN and hole density the DTA units; however, as with **4TABN1**, strong overlap between hole and electron densities are apparent on the phenyl ring on the BN. It is the strong overlap here which results in excited states with mixed CT/LE character for the majority of the excited states, with T_4 of **5TABN2** being LE in nature. A cross comparison of the calculations using the M062X functional, which contains a greater degree of Hartree Fock exchange, was performed and the results obtained are nearly identical to those with PBE0. See below.

Table **S6**. Ground state properties of each emitter at M062X/6-31G(d,p) level.

**Compound**	**HOMO / eV**	**LUMO / eV**	**Δ*E* / eV**
**4TABN1**	-6.14	-0.26	5.88
**5TABN2**	-6.29	-0.63	5.66
**6TABN3**	-6.29	-0.73	5.56

Table **S7**. Excited state properties of each emitter at their lowest energy ground state conformation at TDA-M06-2X/6-31G(d,p).

**Compound**	**S_1 (φ_S) / eV**	***f***	**T_1 (φ_S) / eV**	**T_2 (φ_S) / eV**	**T_3 (φ_S) / eV**	**T_4 (φ_S) / eV**	**ΔE_{ST} / eV**
**4TABN1**	3.78 (0.59)	0.37	3.33 (0.64)	3.39 (0.79)	3.80 (0.83)	3.89 (0.82)	0.46
**5TABN2**	3.64 (0.56)	0.51	3.28 (0.61)	3.35 (0.71)	3.36 (0.74)	3.71 (0.79)	0.36
**6TABN3**	3.61 (0.57)	0.73	3.26 (0.64)	3.31 (0.68)	3.34 (0.68)	3.37 (0.68)	0.35

Where φ_S is an indication of overlap calculated using the NANCYX 2.0 package, with a value of 1 = locally excited (LE) nature and a value of 0 = charge transfer (CT).

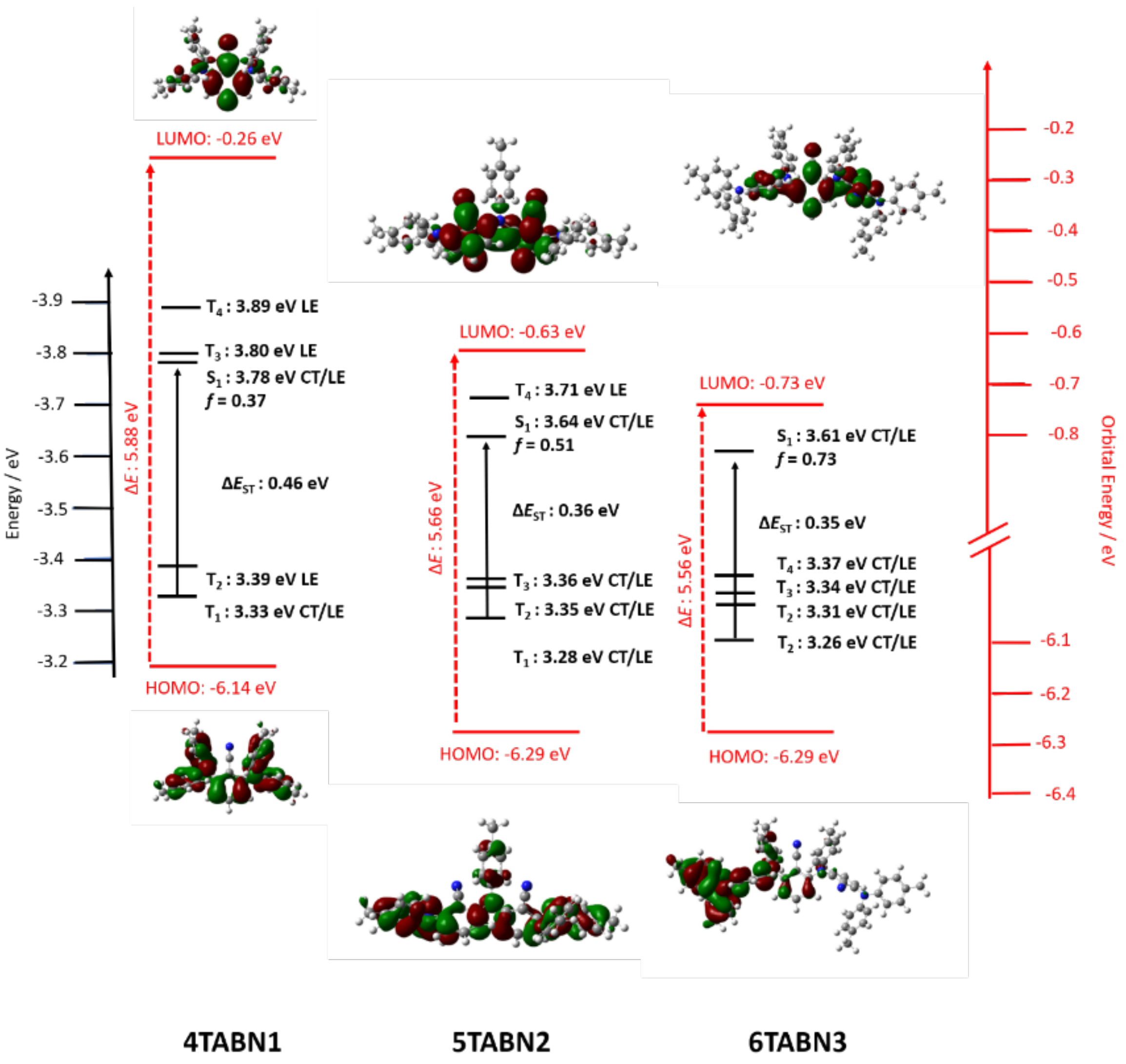

Figure **S38**. HOMO LUMO plots and energies along with the vertical excited state energies calculated at TDA-M06-2X 6-31G(d,p).

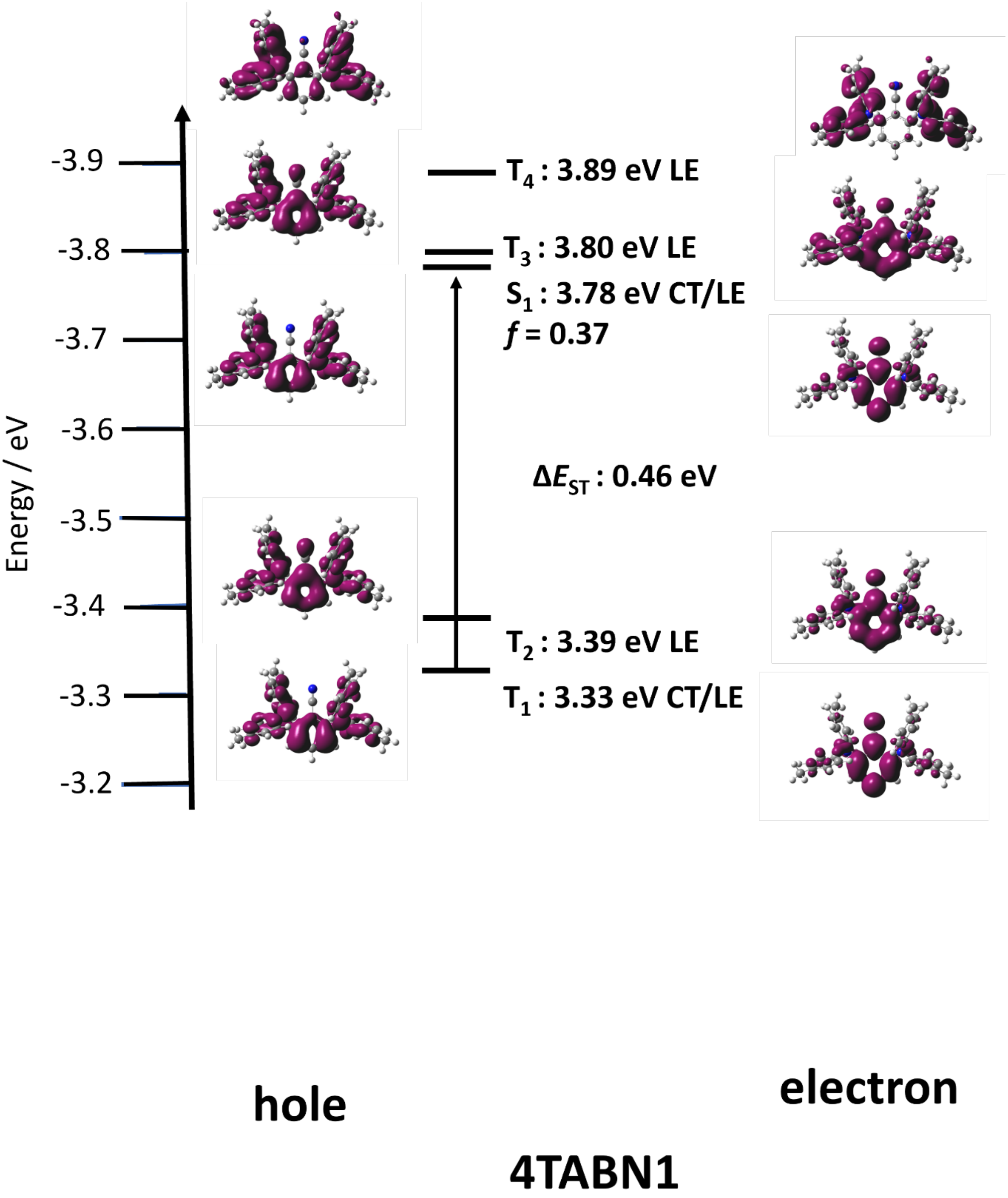

Figure **S39**. Hole/electron plots of **4TABN1** calculated at TDA-M06-2X/6-31G(d,p) using the attachment detachment formalism, where *f* = oscillator strength.

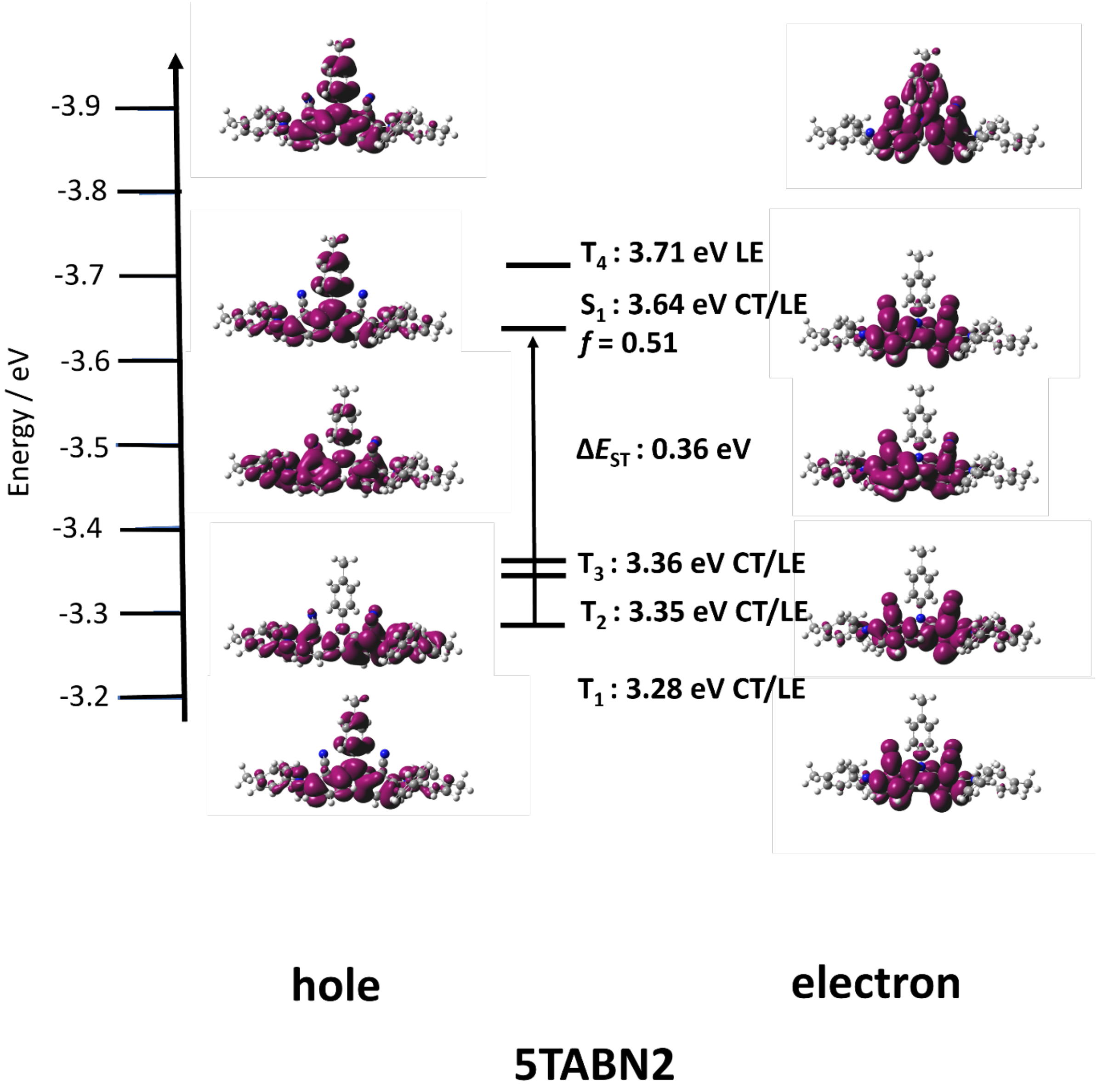

Figure **S40**. Hole/electron plots of **5TABN2** calculated at TDA-M06-2X/6-31G(d,p) using the attachment detachment formalism, where *f* = oscillator strength.

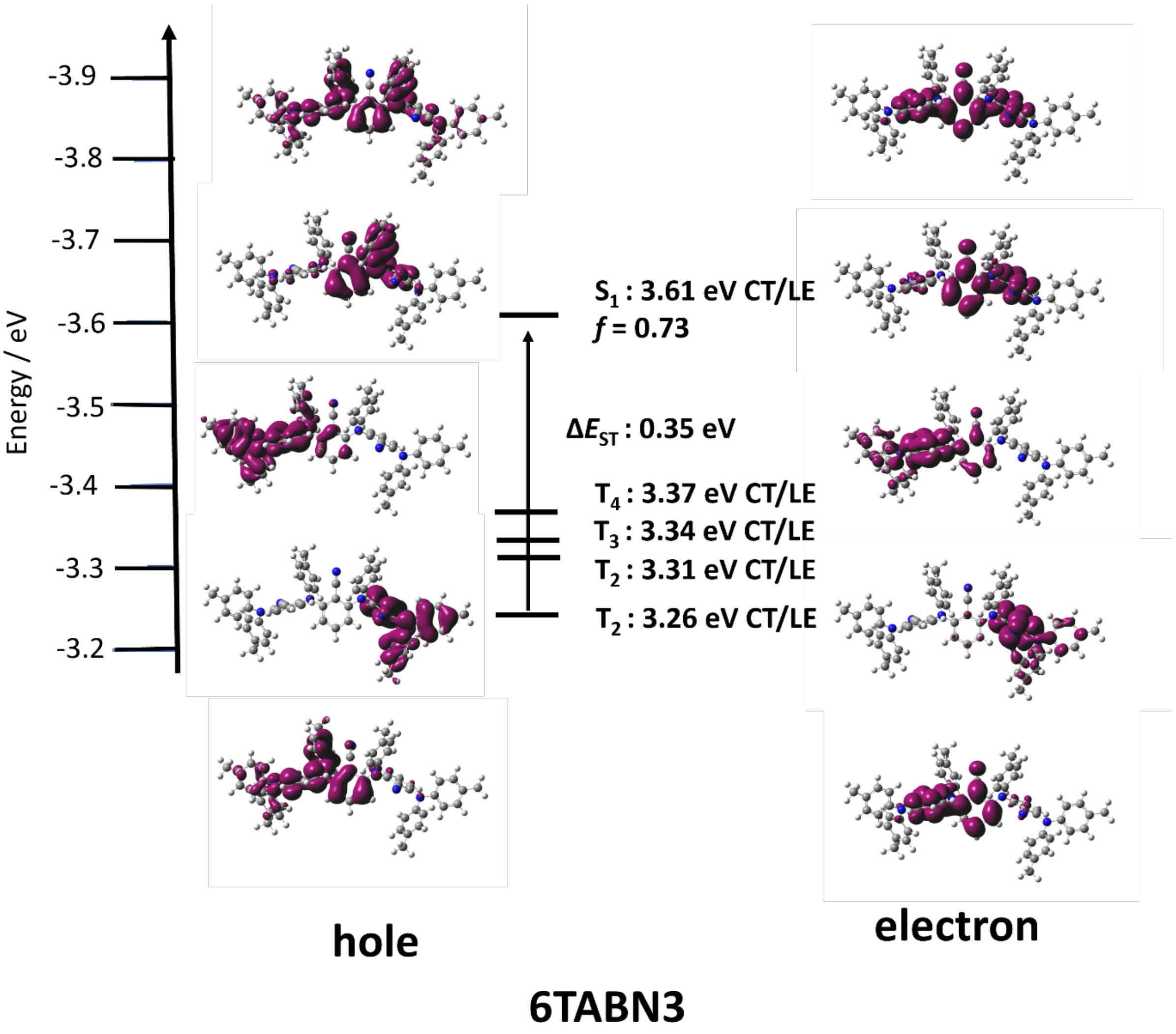

Figure **S41**. Hole/electron plots of **6TABN3** calculated at TDA-M06-2X/6-31G(d,p) using the attachment detachment formalism, where *f* = oscillator strength.

PBE0/M062X cross-comparison

Interestingly, when comparing calculations conducted with the PBE0 to M062X functionals, nearly identical trends in terms of orbital energies and excited state energies across the series are observed; further, the assigned nature of the excited states is identical between the two functionals. The trend in HOMO and LUMO values likewise follow the same trend, with a destabilized HOMO and stabilized LUMO culminating in a decreasing Δ*E* from **4TABN1** to **6TABN3** (Tables **S2** and **S3**). The most striking observation is related to the natures and energies of the excited states. Usually for PBE0 there is an overstabilization of CT excited states,[12] which is much less pronounced when using M062X, as it has a

greater percentage of Hartree-Fock contribution (25% in PBE0 and 56% in M062X).[13] As the excited states in these compounds are primarily of CT/LE character, calculations using either functional predict effectively the same nature for each of the S_1 and T_{1-4} excited states (Figures **S34** and **S36**). Further, the number of predicted intermediate triplet states reported is identical. The data related to PBE0 are found in Tables **S2** and **S4** while those relating to M062X are found in Tables **S3** and **S7**.

Table S8. Selected crystallographic data.

	**4TABN**	**5TABN2**
Formula	$C_{35}H_{31}N_3$	$C_{49}H_{41}N_5$
fw	493.63	699.87
crystal description	Yellow needle	Yellow needle
crystal size [mm^3]	0.32×0.02×0.02	0.11×0.03×0.01
space group	*Pnma*	*C*2/*c*
a [Å]	5.9765(3)	50.7238(11)
b [Å]	20.0083(10)	5.7920(2)
c [Å]	27.4527(13)	29.0379(7)
β [°]		90.522(2)
vol [Å]^3	3282.8(3)	8530.8(4)
Z	4	8
ρ (calc) [g/cm^3]	0.999	1.090
μ [mm^{-1}]	0.059	0.497
F(000)	1048	2960
reflections collected	39670	42597
independent reflections (R_{int})	4065 (0.0658)	7650 (0.0854)
parameters, restraints	180, 0	492, 0
GoF on F^2	1.033	1.019
R_1 [$I > 2\sigma(I)$]	0.0470	0.0462
wR_2 (all data)	0.1133	0.1241
largest diff. peak/hole [e/Å^3]	0.152, -0.190	0.194, -0.214

References

1. Pavlishchuk, V. V.; Addison, A. W., Conversion constants for redox potentials measured versus different reference electrodes in acetonitrile solutions at 25°C. *Inorg. Chim. Acta* **2000,** *298*, 97-102.
2. Cardona, C. M.; Li, W.; Kaifer, A. E.; Stockdale, D.; Bazan, G. C., Electrochemical Considerations for Determining Absolute Frontier Orbital Energy Levels of Conjugated Polymers for Solar Cell Applications. *Adv. Mater.* **2011,** *23*, 2367-2371.
3. Baleizão, C.; Berberan-Santos, M. N., Thermally activated delayed fluorescence as a cycling process between excited singlet and triplet states: Application to the fullerenes. *J. Chem. Phys.* **2007,** *126*, 204510.
4. Dias, F. B.; Penfold, T. J.; Monkman, A. P., Photophysics of thermally activated delayed fluorescence molecules. *Method. Appl. Fluoresc.* **2017,** *5*, 012001.
5. G. W. T. M. J. Frisch, H. B. S., G. E. Scuseria, M. A. Robb, J. R. Cheeseman, G. Scalmani, V. Barone, B. Mennucci, G. A. Petersson, H. Nakatsuji, M. Caricato, X. Li, H. P. Hratchian, A. F. Izmaylov, J. Bloino, G. Zheng, J. L. Sonnenberg, M. Hada, M. Ehara, K. Toyota, R. Fukuda, J. Hasegawa, M. Ishida, T. Nakajima, Y. Honda, O. Kitao, H. Nakai, T. Vreven, J. A. Montgomery Jr., J. E. Peralta, F. Ogliaro, M. Bearpark, J. J. Heyd, E. Brothers, K. N. Kudin, V. N. Staroverov, R. Kobayashi, J. Normand, K. Raghavachari, A. Rendell, J. C. Burant, S. S. Iyengar, J. Tomasi, M. Cossi, N. Rega, J. M. Millam, M. Klene, J. E. Knox, J. B. Cross, V. Bakken, C. Adamo, J. Jaramillo, R. Gomperts, R. E. Stratmann, O. Yazyev, A. J. Austin, R. Cammi, C. Pomelli, J. W. Ochterski, R. L. Martin, K. Morokuma, V. G. Zakrzewski, G. A. Voth, P. Salvador, J. J. Dannenberg, S. Dapprich, A. D. Daniels, Ö. Farkas, J. B. Foresman, J. V. Ortiz, J. Cioslowski, D. J. Fox, , Gaussian Inc., Wallingford, CT, 2013.
6. Adamo, C.; Barone, V., Toward reliable density functional methods without adjustable parameters: The PBE0 model. *J. Chem. Phys.* **1999,** *110*, 6158-6170.
7. Dunning, T. H., Gaussian basis sets for use in correlated molecular calculations. I. The atoms boron through neon and hydrogen. *J. Chem. Phys.* **1989,** *90*, 1007-1023.
8. Zhao, Y.; Truhlar, D. G., The M06 suite of density functionals for main group thermochemistry, thermochemical kinetics, noncovalent interactions, excited states, and transition elements: two new functionals and systematic testing of four M06-class functionals and 12 other functionals. *Theor. Chem. Acc.* **2007,** *120*, 215-241.
9. Hirata, S.; Head-Gordon, M., Time-dependent density functional theory within the Tamm–Dancoff approximation. *Chem. Phys. Lett.* **1999,** *314*, 291-299.
10. Etienne, T.; Assfeld, X.; Monari, A., Toward a Quantitative Assessment of Electronic Transitions' Charge-Transfer Character. *J. Chem. Theory Comput.* **2014,** *10*, 3896-3905.
11. R. K. Dennington, T. M., J, KS, Semichem Inc.: Shawnee Mission, 2019.
12. Moral, M.; Muccioli, L.; Son, W. J.; Olivier, Y.; Sancho-García, J. C., Theoretical Rationalization of the Singlet–Triplet Gap in OLEDs Materials: Impact of Charge-Transfer Character. *J. Chem. Theory Comput.* **2014,** *11*, 168-177.
13. Sun, H.; Zhong, C.; Brédas, J.-L., Reliable Prediction with Tuned Range-Separated Functionals of the Singlet–Triplet Gap in Organic Emitters for Thermally Activated Delayed Fluorescence. *J. Chem. Theory Comput.* **2015,** *11*, 3851-3858.
